# Supplementary material for: SF3B1 hotspot mutations confer sensitivity to PARP inhibition by eliciting a defective replication stress response
Source: Nat Genet. 2023 Jul 31;55(8):1311–23. doi: 10.1038/s41588-023-01460-5 (PMC10412459; doi:10.1038/s41588-023-01460-5)

nature genetics

Article

https://doi.org/10.1038/s41588-023-01460-5

SF3B1 hotspot mutations confer sensitivity to PARP inhibition by eliciting a defective replication stress response

In the format provided by the authors and unedited

Supplementary Information

- Supplementary methods
- Supplementary Information Figures 1-7
- Uncropped gels Supplementary Information Figures 4 and 6

Additional Methods

RNA extraction, PCR, RT-PCR and Sanger sequencing.

RNA was extracted using Trizol (Life Technologies) according to the manufacturer's instructions. Retention of engineered alterations was performed by PCR and RT-PCR using KAPA Taq DNA polymerase (KAPA Biosystems) and Superscript III (Invitrogen), with 50ng of RNA per reaction mixture. Samples were Sanger sequenced[8] and visualised using Chromas (https://technelysium.com.au/wp/chromas/).

Chemicals.

Talazoparib, niraparib, olaparib, VX-970, AZD0156 and KU-55933, CCT245737, CCT2424737, XL413 (BMS863233), cycloheximide and MG-132 were purchased from Selleck Chemicals (Stratech).

Western blotting.

Whole-cell protein extracts from cell lines and patient samples (PiCCLe trial) were prepared from cells lysed with nuclear lysis buffer (10mM HEPES pH 7.4, 350mM NaCl, 1mM MgCl2, 1% Triton X-100, 10% Glycerol) enhanced with protease inhibitor and phosphatase inhibitor cocktail tablets (Roche, cOmplete PIC) as well as Benzonase® Nuclease (Millipore) diluted 1/1000. Cytosolic fractions were isolated with cytosol buffer (10mM HEPES pH 7.4, 10mM NaCl, 3mM MgCl2, 0.5% NP40) supplemented with protease inhibitor and phosphatase inhibitor cocktail tablets. Remaining nuclear fractions were prepared with nuclear lysis buffer. Protein concentrations were quantified using the colorimetric Bio-Rad Protein Assay (Bio-Rad). 20 µg of cell lysate was loaded on NuPage 4-12% gradient Bis-Tris gels (Invitrogen) and proteins were separated using SDS-PAGE at 160V for 90 minutes. Proteins were transferred on to a PVDF membrane and blocked in 5% milk in TBS-T (0.1% Tween-20 in TBS) for 60 minutes. The membrane was incubated overnight in primary antibody at 4°C. The membrane was then washed three times in TBS-T before a 60-minute incubation with HRP-conjugated secondary antibody. The membrane was then washed three times in TBS-T. Target protein abundance was detected using the SuperSignal™ West Pico PLUS Chemiluminescent Substrate (ThermoFisher) and an X-ray developing system. Protein expression was quantified using ImageJ. Note samples from the PiCCLe trial were exposed to 50nM talazoparib for 48 hours before western blot to mimic the conditions where CINP was identified from the Mass Spectrometry analysis of MEL202 isogenic cells.

Genome-wide CRISPR screen in K562 cells.

Doxycycline inducible K562-Cas9 cell lines were generated through the transduction of the $K562^{K700E}$ cells with an Edit-R Inducible Lentiviral hEF1a-Blast-Cas9 Nuclease and

subsequent selection in 10 μg/ml blasticidin. Cells were infected with a multiplicity of infection (MOI) of 0.2, with a previously validated human lentiviral gRNA CRISPR library (Addgene #67989)[36,62]. Cell lines underwent puromycin selection followed by talazoparib selection at 100nM for 24 days. DNA was extracted from surviving cells using the DNeasy Blood and Tissue Kit (Qiagen) as per manufacturers' instructions. sgRNA sequences were amplified with Q5 polymerase (NEB) with specific primers (Table S9) and sequenced on the Illumina HiSeq2500[36]. Raw read counts were converted to parts per ten million (pptm) and $\log_2$ transformed (after adding a pseudo count of 0.5) and median viability effect z-scores (the rate of decrease in abundance of each sgRNA in the population over time in the absence of drug treatment) calculated for all guides covering a gene and ranked according to z-score[36].

Splice variant analysis by qPCR.

The analysis of alternatively spliced exons was performed using 384-well plates using SYBR Green reagents (Invitrogen) on an ABI 9700HT. Primers were designed (http://primer3.ut.ee) for sequences of predicted, conical and alternative spliced exons[3]. Relative abundance of spliced and un-spliced transcript was defined by $2^{-(\text{CT variant1 mRNA} - \text{CT variant2 mRNA})}$ and statistical significance was evaluated through a two-tailed Mann-Whitney U-test for each splicing event. Primers are listed in **Table S8**.

***Ex vivo* talazoparib efficacy studies.**

The efficacy of talazoparib treatment on organoid models (*ex vivo,* 3D Matrigel assay) for the selected PDO models, SUM149 cell lines and the subsequent PDX11310 treatment *in vivo* study was carried out by Crown Bioscience San Diego. Briefly, 10 NOD-SCID mice were inoculated with each of the PDX models and tumours between 500-800 mm^3 were harvested and transferred on ice immediately. Tumours were processed using MACS tumour dissociation kit (Cat no 130-095-929) according to the manufacturer's protocol. Cells were seeded in 3D Matrigel assays in which cells were treated with the compounds at 9 doses in triplicate. Each model was tested with test articles and a reference drug control (cisplatin). Viability was measured using Cell Titre Glo after 7 days of drug exposure.

Immunohistochemistry

4μm FFPE sections of FFPE blocks of PDX models and fixed cell line pellets (10% neutral buffered formalin (NBF) for 12 hrs) were mounted on X-tra™ Adhesive slides (Leica Biosystems). IHC for pericentrin was performed on the automated Ventana (Discovery, Roche) platform with CC1 antigen retrieval and UltraMap anti-Rabbit HRP kit using anti-PCNT rabbit polyclonal (HPA 016820 Sigma) AB (1:100). CINP IHC was performed using the rabbit monoclonal Anti-CINP antibody [EPR14446] ab180955 (Abcam) at 1:400 dilution with high pH9 microwave antigen retrieval and 1hr room temperature antibody incubation. $\text{MEL202}^{\text{R625G-}}$

^{DEG} parental cells or MEL202$^{R625G-DEG}$ cells transfected with siRNA targeting *CINP* were used as positive and negative controls respectively. Bound antibody was visualized with 3,3′ -diaminobenzidine (DAB+) (Dako). Digital images were analysed using QPath software v.0.3.0[62] assessing nuclear and cytoplasmic average intensity across the sections. Individual PCNT events were assessed by surface area (μm^2) and mean intensity (MI, values of grey: 0 to 255)[63]

Supplementary Information Figure 1-
related to Figure 1

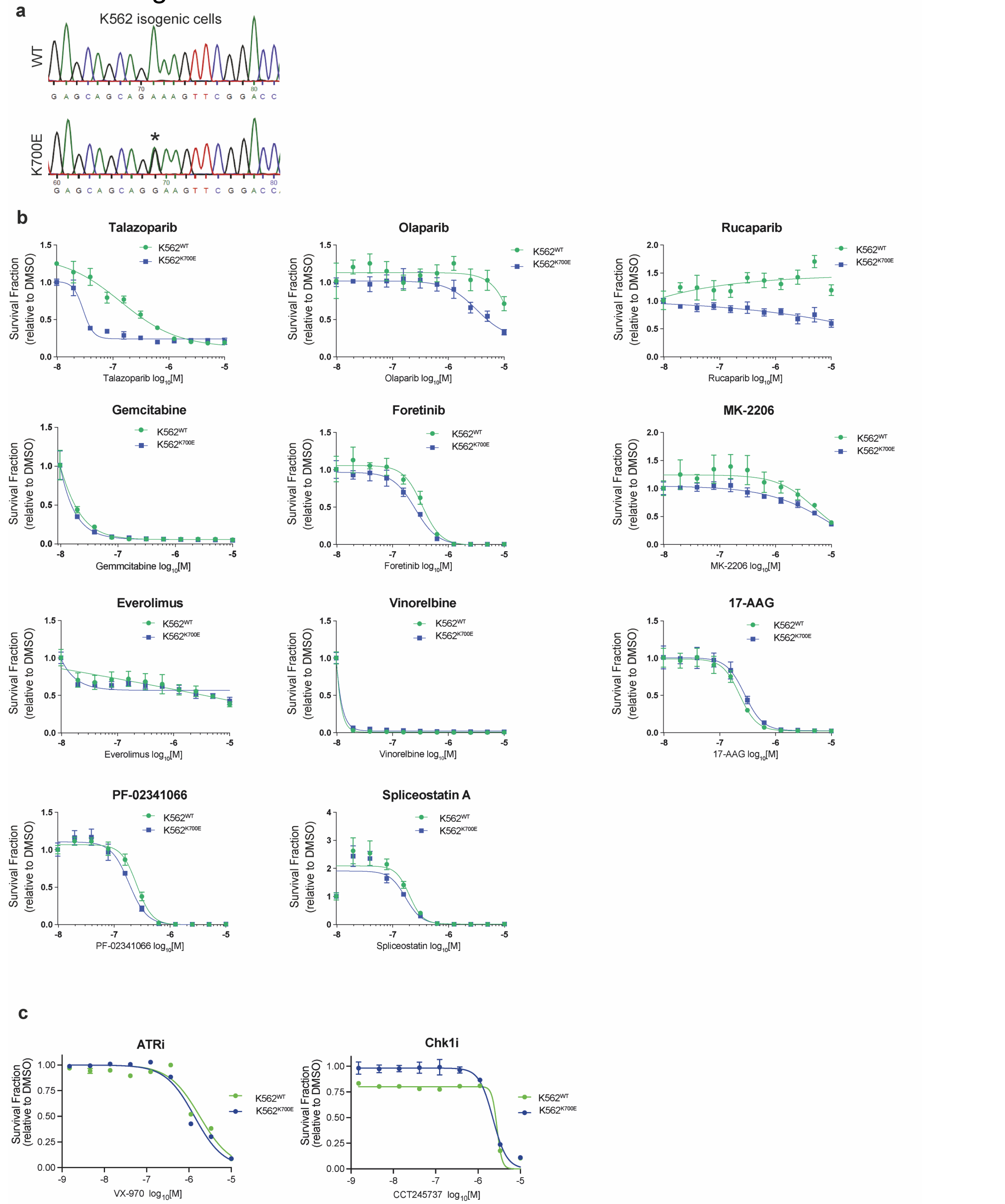

Supplementary Information Figure 1. Drug screen validation

a, Sequencing chromatogram of K562 isogenic cell lines that express wild-type (WT) or mutant (K700E) *SF3B1*. **b,** 5 day dose response curves of isogenic K562 SF3B1^{WT} and SF3B1^{K700E} cells treated with indicated agents identified as hits from the initial drug screen (*n*=1 biological replicate. Data are presented as mean ± s.e.m of at least *n*=3 technical replicates. **c,** 5 day dose response curves of isogenic K562 SF3B1^{WT} and SF3B1^{K700E} cells treated with the ATRi VX-970 and CHK1i (CCT245737), Data are presented as mean ± s.e.m of at least *n*=3 technical replicates.

Supplementary Information Figure 2 - related to Figure 2

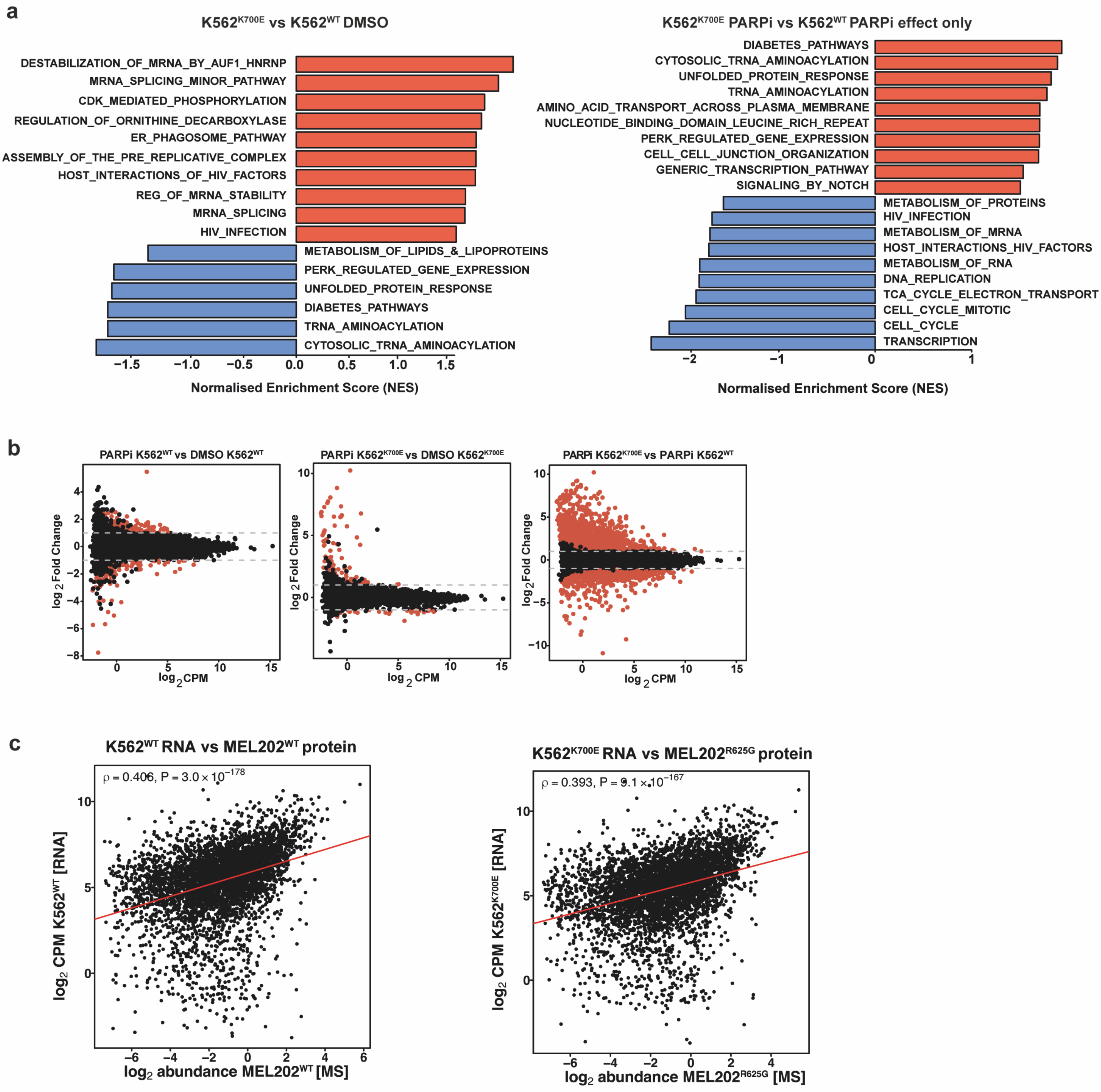

Supplementary Information Figure 2. *SF3B1* hotspot mutations confer transcriptional alterations. **a,** Barplots of normalised enrichment score of enriched pathways from the Gene Set enrichment analysis (GSEA) of K562^{WT} and K562^{K700E} RNA sequencing data +/- talazoparib exposure depicting PARPi effect only (K562^{K700E} PARPi – K562^{WT} PARPi) - (K562^{K700E} DMSO – K562^{WT} DMSO). **b,** MA plots highlighting the significantly differentially expressed genes between the highlighted comparisons in the K562 RNA-sequencing data. Significantly differentially expressed genes are depicted in red (FDR < 0.01, |LFC| > 1). **c,** Scatter plots showing the correlation between K562 RNA-sequencing and MEL202 proteomics data.

Supplementary Information Figure 2 - related to Figure 2

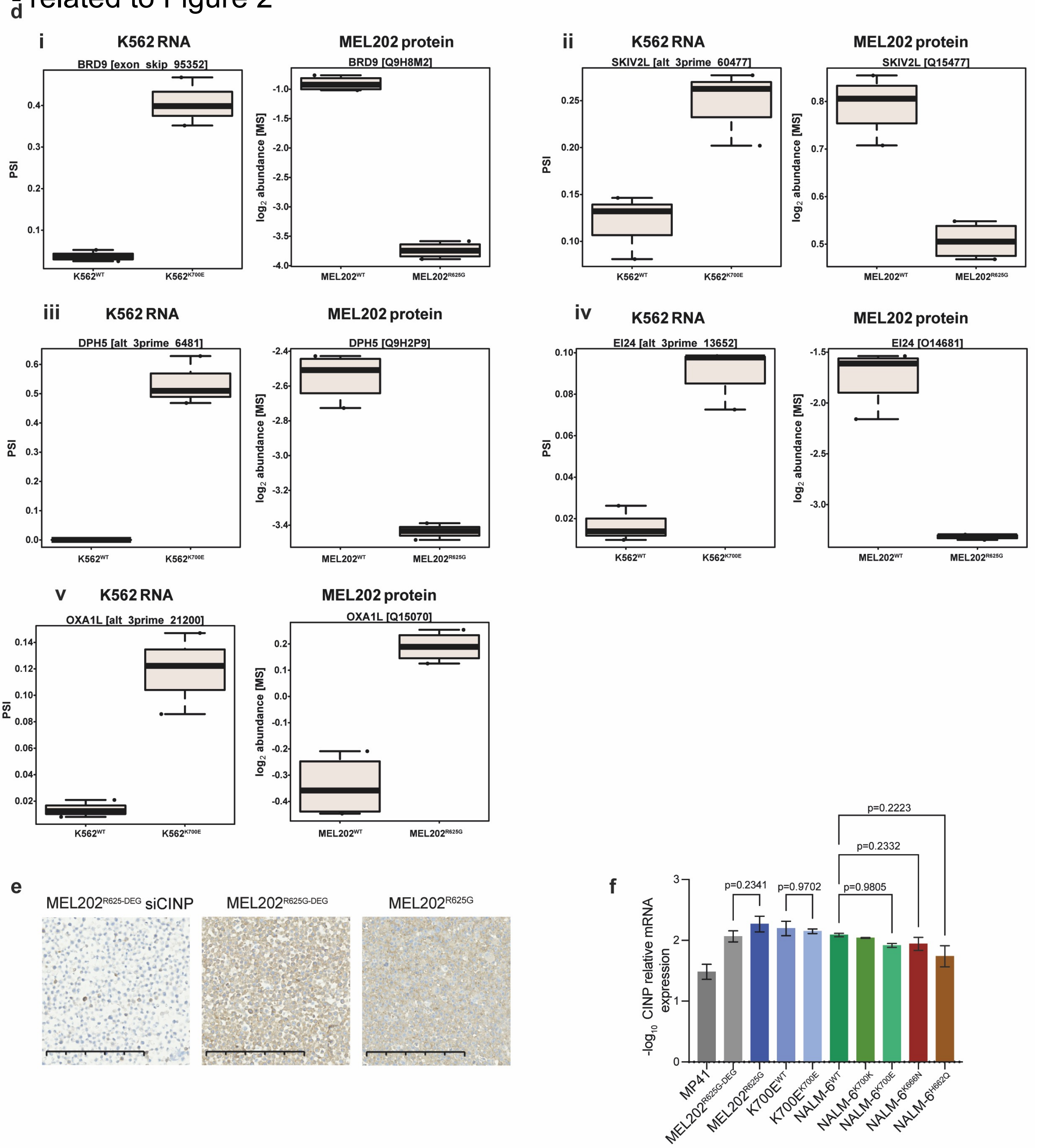

Supplementary Information Figure 2. *SF3B1* hotspot mutations confer transcriptional alterations.

d, Boxplots of percent spliced in (PSI) ratio in K562 RNA-sequencing data of the specific alternatively spliced junction of transcripts that are consistently aberrantly spliced in multiple patient data sets between *SF3B1*^{WT} and *SF3B1*^{MUT} primary tumours[1] and the $\log_2$ protein abundance identified from the mass-spectrometry data from MEL202 isogenic cell line model. i) BRD9, ii) SKIV2L, iii) DPH5, iv) EI24 and v) OXA1L. data are from n=3 independent biological repeats. **e**, Representative micrographs of CINP immunohistochemistry of MEL202$^{R625G\text{-}DEG}$ siCINP, MEL202$^{R625G\text{-}DEG}$ and MEL202^{R625G} paraffin embedded cell pellets (scale bar = 200μm). Images are representative of at least n=4 independent biological replicate experiments. **f,** Barplot showing *CINP* RNA levels in the MP41 SF3B1^{WT} UM cells and MEL202, K562 and NALM-6 series of isogenic cell lines, normalised to β-Actin. Pair-wise t-tests with Tukey's multiple correction. Data are mean +/- s.e.m. of n=4 technical replicates.

Supplementary Information Figure 4 - related to Figure 4

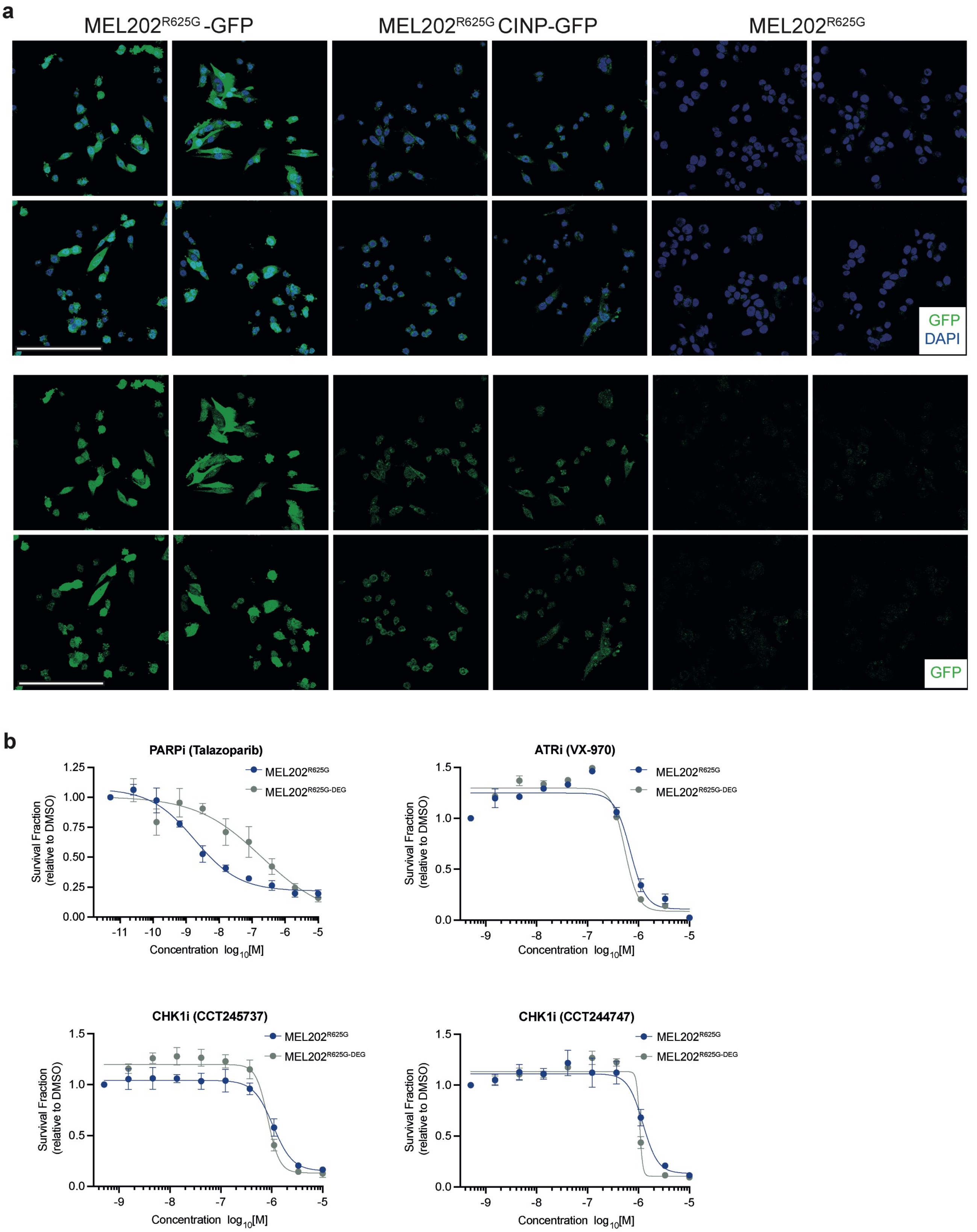

Supplementary Information Figure 4. *CINP* re-expression rescues PARPi sensitivity in *SF3B1* mutant cells.

a, Representative immunofluorescence images showing green fluorescence protein (GFP) fluorescence intensity in MEL202^{R625G} cells expressing control-GFP or CINP-GFP, or untransfected control cells (Scale bar = 100μm). **b,** 5 day talazoparib, ATRi (VX-970), CHK1i (CCT245737 and CCT244747) dose-response curves showing the survival fraction, relative to DMSO, of MEL202 isogenic cells. Data are presented as mean ± s.d. of *n*=3 technical replicates.

Supplementary Information Figure 5 - related to Figure 5

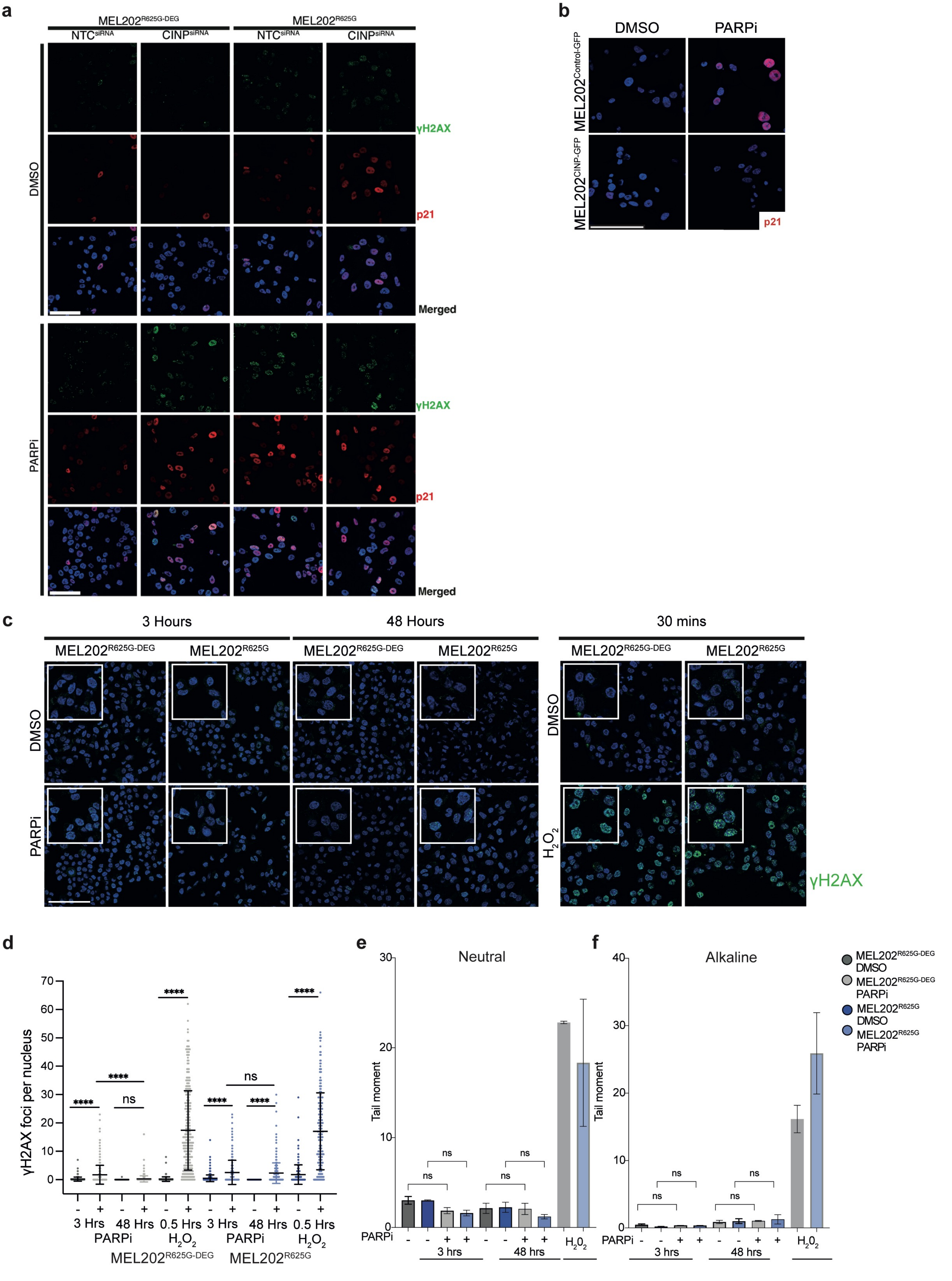

Supplementary Information Figure 5 - related to Figure 5

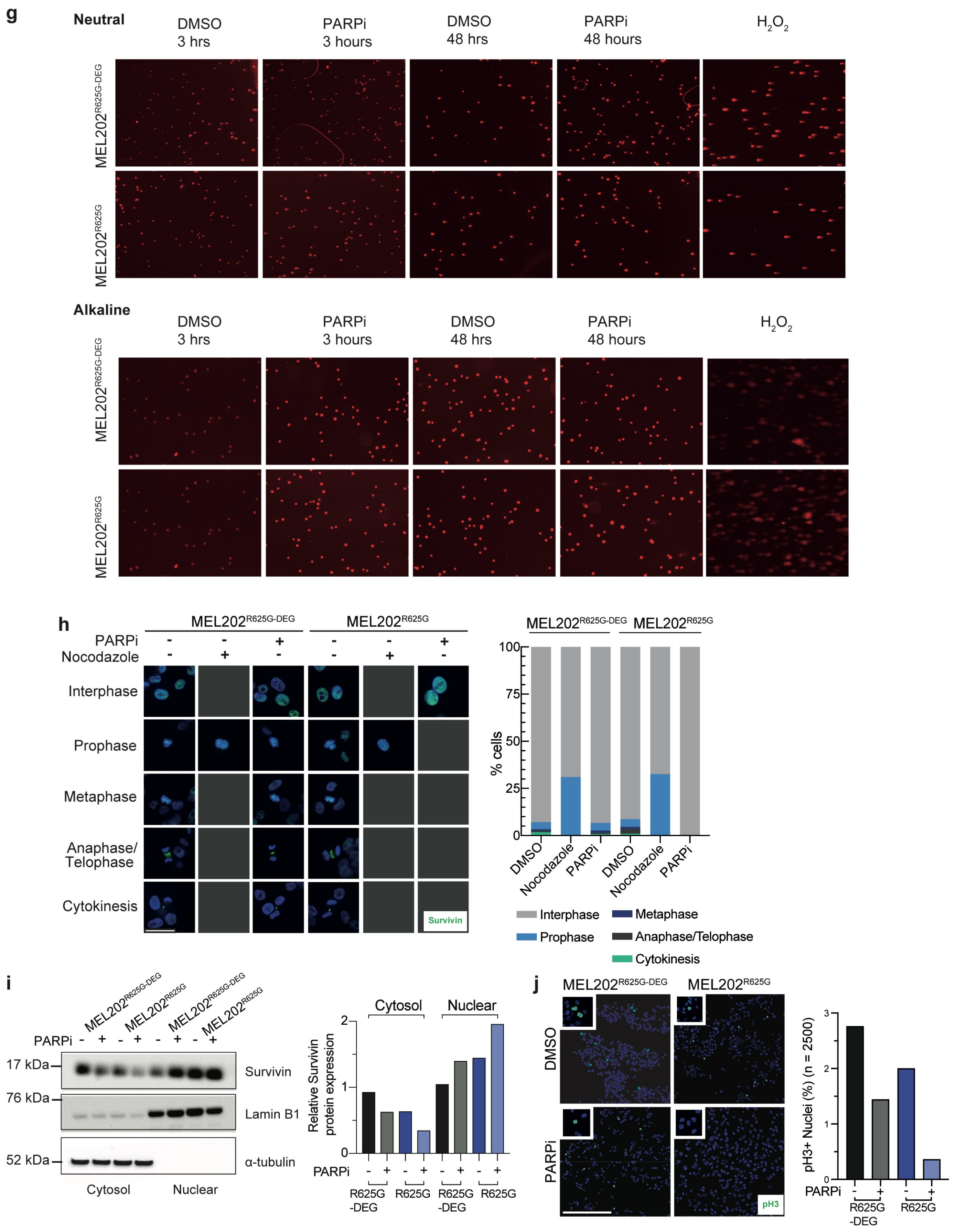

Supplementary Information Figure 5. Cell cycle arrest at the G_2/M checkpoint after PARPi exposure.

a, Representative immunofluorescence images to corresponding Extended Data Figure 9 g-h, showing the nuclear intensity of p21 and γH2AX in MEL202 isogenic pair after NTC or *CINP* gene silencing and 48 hours of 50 nM talazoparib or DMSO exposure (Scale bar = 100 μm). **b-c,** Representative immunofluorescent (b) images and (c), quantification of γH2AX in $MEL202^{R625G-DEG}$ and $MEL202^{R625G}$ cells after 3 hours and 48 hours talazoparib and 30 minutes H_2O_2 control exposure (Scale bar = 100 μm). **d-e,** Quantification and representative images (f) from the neutral (d) and alkaline (e) COMET assay for detection of double strand breaks and quantification (tail moments in $MEL202^{R625G-DEG}$ and $MEL202^{R625G}$ cells after 3 hours and 48 hours talazoparib and DMSO and 30 minutes H_2O_2 control). COMETs were quantified using the COMETScore software package (TriTek Corporation, Sumerduck, VA). Experiments were performed *n*=3 biological replicates for PARPi and *n*=2 biological replicates for H_2O_2 control exposure and a minimum of 100 COMETs analysed per replicate slide. Note the COMET assays were run independently for the indicated timepoints due to the nature and number of replicates in the assay. Hence, DMSO controls were included for each time point**. g,** Representative immunofluorescence images showing the nuclear intensity of p21 in $MEL202^{R625G}$ cells expressing control-GFP or CINP-GFP, after 48 hours of 50 nM talazoparib or DMSO exposure (Scale bar = 100 μm). **h**, Representative immunofluorescence images and corresponding stacked bar plots showing the percentage of MEL202 isogenic cells in each phase of mitosis after 48 hours of 50 nM talazoparib, 1 μg/ml nocodazole, or DMSO exposure. Data are of *n*=1 biological replicate (Scale bar = 10 μm). **i**, Western blot and corresponding column bar graph showing cytosolic and nuclear survivin expression in MEL202 isogenic cells after 48 hours of 50 nM talazoparib or DMSO exposure. Cytosolic and nuclear survivin expression are normalised to α-tubulin and Lamin B1 expression respectively. **j**, Representative immunofluorescence images and corresponding column bar graph showing the percentage of phospho-histone H3 (Ser10) positive (pH3+) $MEL202^{R625G-DEG}$ and $MEL202^{R625G}$ isogenic cells after 48 hours of 50 nM talazoparib exposure, scale bar = 250 μm. Data are of *n*=1 biological replicate, error bars show ± s.d.

Supplementary Information Figure 6 - related to Figure 6

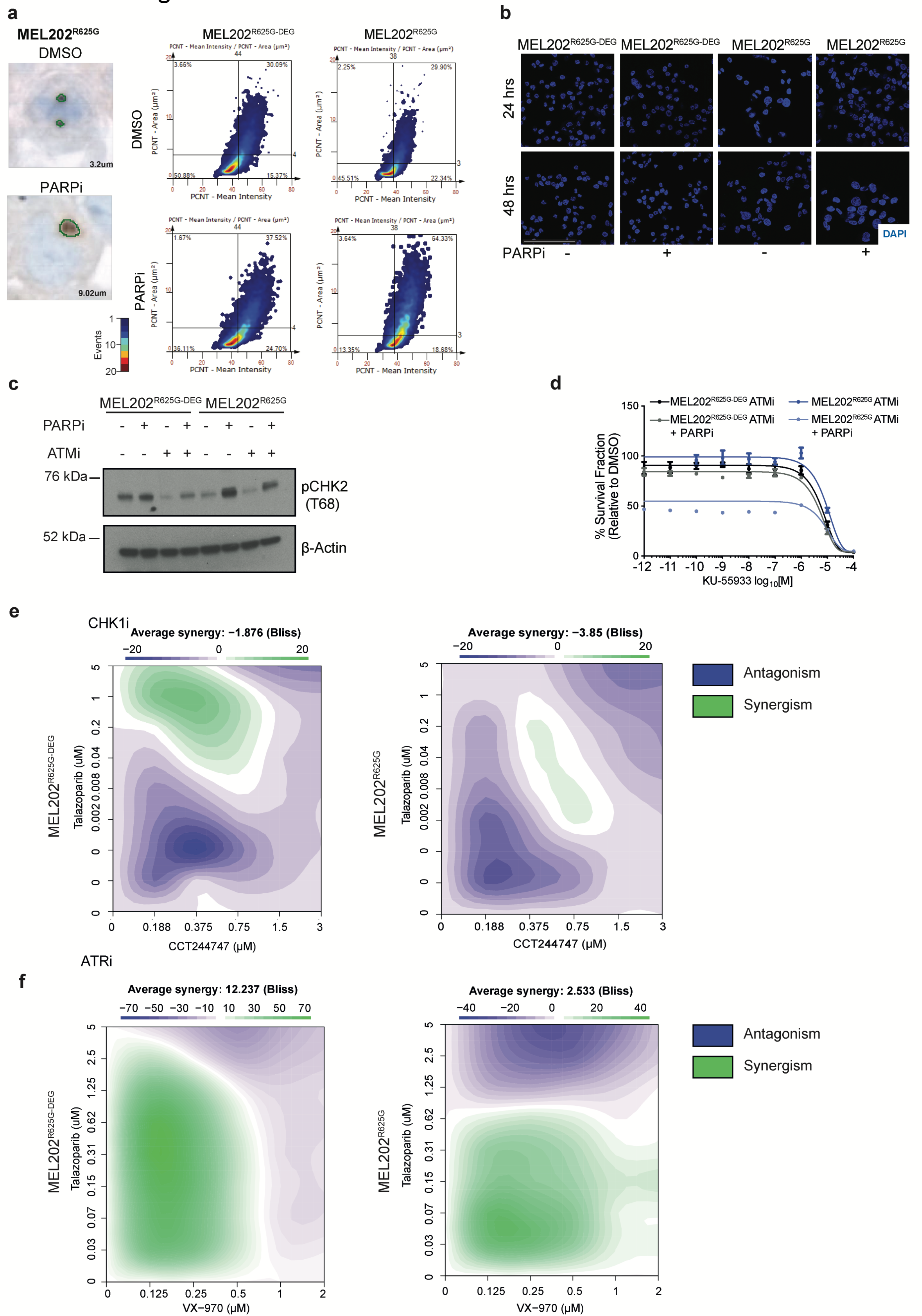

Supplementary Information Figure 6. PARPi and ATMi synergise in *SF3B1* mutant cells.
a, Representative images, and scatter plot quantification of pericentrin mean intensity/area in MEL202$^{R625G\text{-}DEG}$ and MEL202^{R625G} xenografted tumours (*n*=4 independent mice) treated with DMSO or PARPi. **b**, Representative immunofluorescence images to corresponding Fig. 6a showing the nuclear area of MEL202$^{R625G\text{-}DEG}$ and MEL202^{R625G} isogenic cells after 24 and 48 hours of 50 nM talazoparib or DMSO exposure. Scale bar = 100 μm. **c**, Western blot of pCHK2 (T68) expression in MEL202 isogenic cells after 48 hours of 500 pM talazoparib, 50 nM AZD0156, combination, or DMSO exposure. **d**, KU-55933 dose-response curves showing the survival fraction, relative to DMSO, of MEL202 isogenic cells after 5 days of exposure to KU-5933 alone or in combination with 50 nM talazoparib. Data are of *n*=1 biological replicate, error bars show $\pm$ s.d. of technical replicates. **e-f**, Heatmaps showing BLISS synergy scores based on the survival fraction, relative to DMSO, of MEL202 isogenic cells after 5 days of exposure with talazoparib in combination with CCT244747 (e) or VX-970 (f).

Supplementary Information Figure 7 - related to Figure 7

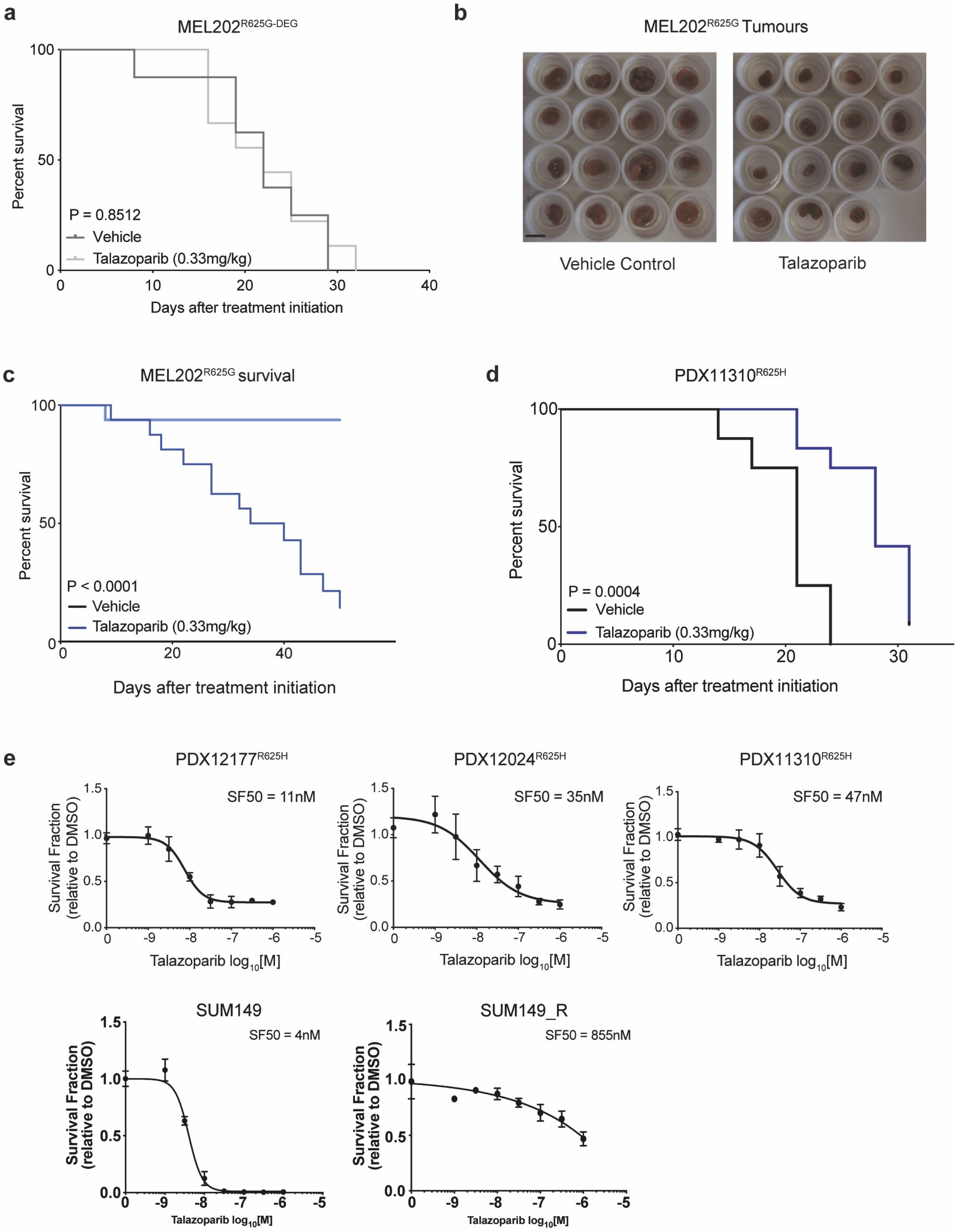

Supplementary Information Figure 7. PARP inhibition suppresses *SF3B1* mutant tumour growth *in vivo*

a, Kaplan-Meier curves of MEL202$^{R625G\text{-}DEG}$ tumours depicting no significant difference in survival in animals treated with talazoparib, (*P*=0.8512, *HR*=1.078, 95% *CI*=0.4146 to 2.80, logrank test). **b,** Images of excised MEL202 xenograft tumours at end-point after undergoing vehicle control or talazoparib treatment (scale bar = 1 cm). **c,** Kaplan-Meier curves of MEL202^{R625G} tumours depicting an increase in survival in animals treated with talazoparib, (*HR*=20.74, 95% *CI*=7.069 to 60.86, logrank test). **d,** Kaplan-Meier curves of PDX11310 tumours depicting an increase in survival in animals treated with talazoparib, (*P*=0.0004, *HR*=3.501, 95% CI = 1.063 to 11.52, logrank test). **e,** Talazoparib, dose-response curves showing the survival fraction, relative to DMSO, of *SF3B1*^{MUT} patient derived xenografts grown in 3D culture and SUM149 *BRCA1*^{MUT} and SUM149 *BRCA1*^{WT} revertant isogenic cells after 5 days of talazoparib treatment. Data are of *n*=1 biological replicate, error bars show $\pm$ s.e.m. of *n*=6 technical replicates.

Uncropped western blot gels
Supplementary information Figure 5i

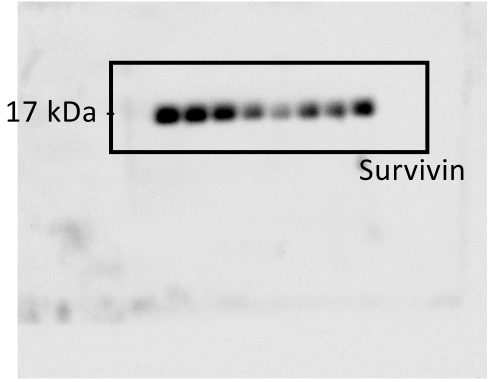

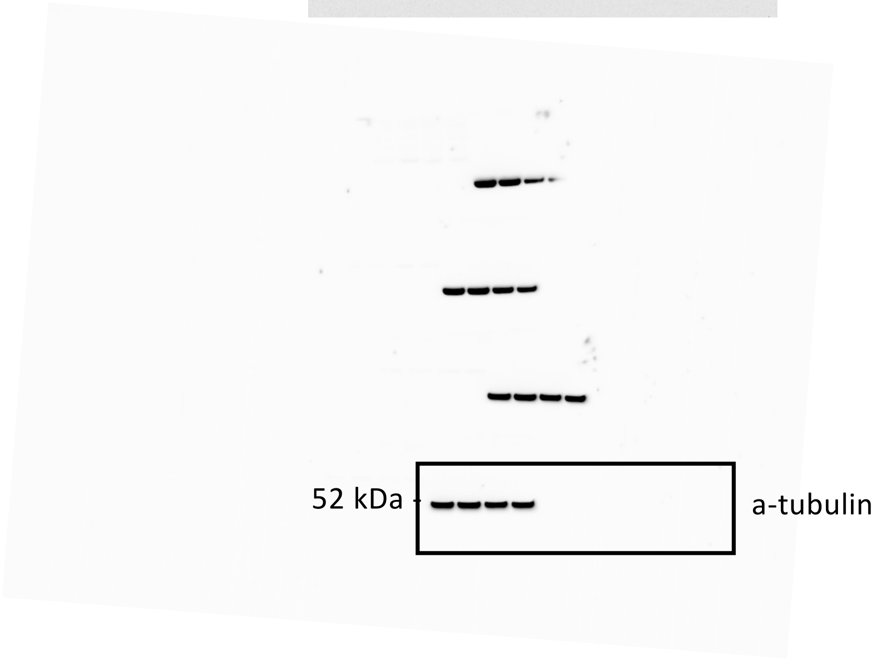

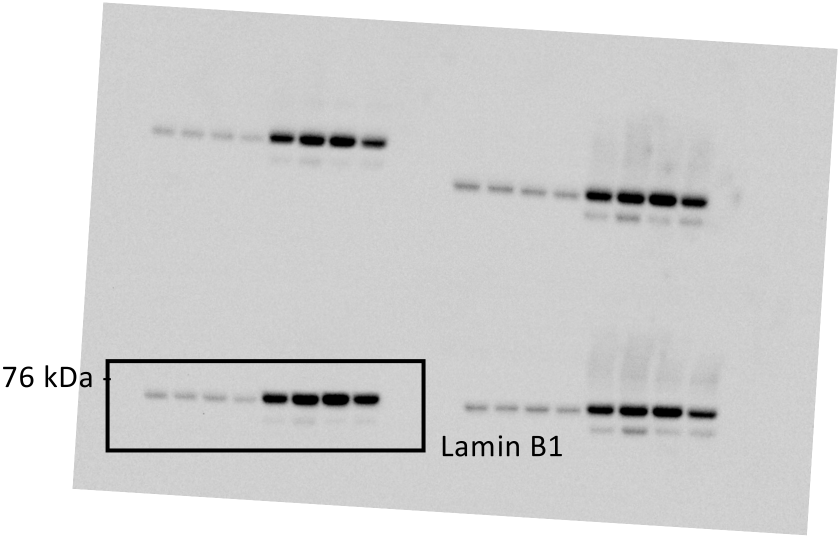

Uncropped western blot gels
Supplementary information Figure 6c

pCHK2 (T68)

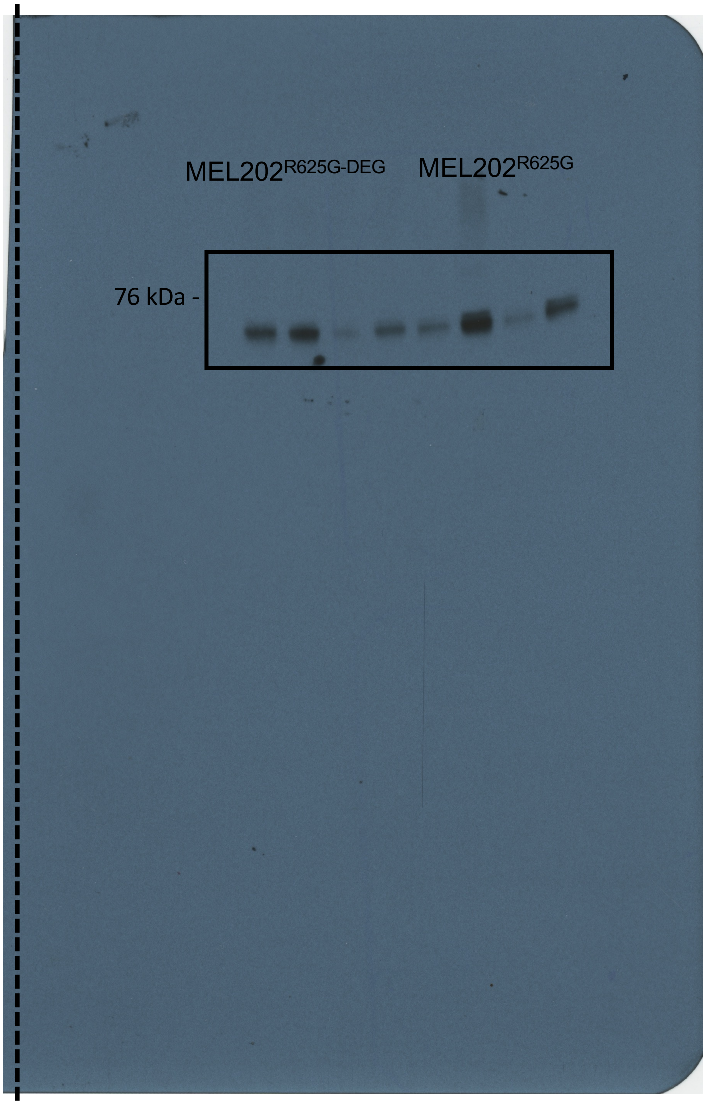

β-actin

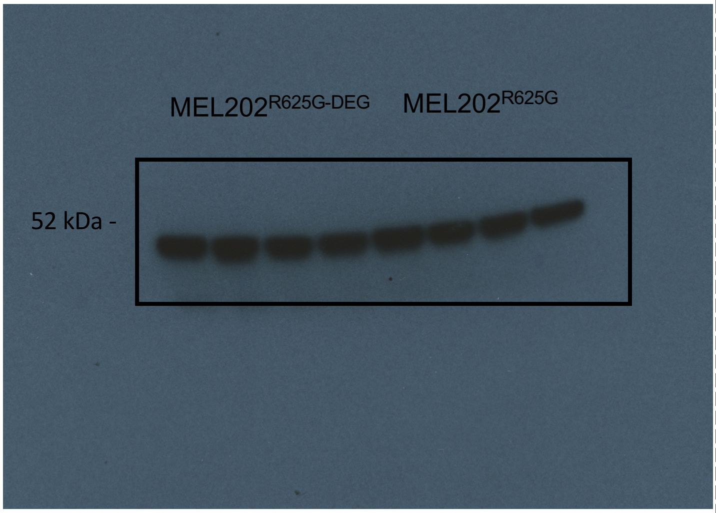

Supplement: Supplementary file 1 — Supplementary Methods, Supplementary Figs. 1–7 and unprocessed blots for Supplementary Figs. 4 and 6. [file 41588_2023_1460_MOESM1_ESM.pdf]
