# Supplementary material for: SF3B1 hotspot mutations confer sensitivity to PARP inhibition by eliciting a defective replication stress response
Source: Nat Genet. 2023 Jul 31;55(8):1311–23. doi: 10.1038/s41588-023-01460-5 (PMC10412459; doi:10.1038/s41588-023-01460-5)
Supplement: Supplementary file 2 — Reporting Summary [file 41588_2023_1460_MOESM2_ESM.pdf]

nature portfolio

Corresponding author(s): Rachael Natrajan

Last updated by author(s): 25th May 2023

Reporting Summary

Nature Portfolio wishes to improve the reproducibility of the work that we publish. This form provides structure for consistency and transparency in reporting. For further information on Nature Portfolio policies, see our Editorial Policies and the Editorial Policy Checklist.

Statistics

For all statistical analyses, confirm that the following items are present in the figure legend, table legend, main text, or Methods section.

n/a	Confirmed	
☐	☒	The exact sample size (*n*) for each experimental group/condition, given as a discrete number and unit of measurement
☐	☒	A statement on whether measurements were taken from distinct samples or whether the same sample was measured repeatedly
☐	☒	The statistical test(s) used AND whether they are one- or two-sided<br>*Only common tests should be described solely by name; describe more complex techniques in the Methods section.*
☐	☒	A description of all covariates tested
☐	☒	A description of any assumptions or corrections, such as tests of normality and adjustment for multiple comparisons
☐	☒	A full description of the statistical parameters including central tendency (e.g. means) or other basic estimates (e.g. regression coefficient) AND variation (e.g. standard deviation) or associated estimates of uncertainty (e.g. confidence intervals)
☐	☒	For null hypothesis testing, the test statistic (e.g. *F*, *t*, *r*) with confidence intervals, effect sizes, degrees of freedom and *P* value noted<br>*Give P values as exact values whenever suitable.*
☒	☐	For Bayesian analysis, information on the choice of priors and Markov chain Monte Carlo settings
☒	☐	For hierarchical and complex designs, identification of the appropriate level for tests and full reporting of outcomes
☐	☒	Estimates of effect sizes (e.g. Cohen's *d*, Pearson's *r*), indicating how they were calculated

Our web collection on statistics for biologists contains articles on many of the points above.

Software and code

Policy information about availability of computer code

Data collection	All histological slides were scanned at x 40 (0.25 µm/pixel) digital magnification using Hamamatsu Nanozoomer XR (Hamamatsu photonics, Hamamatsu, Japan). Digital images in .ndpi format were submitted for quantitative image analysis using HistoQuest 6.0 (Tissugnostic, Vienna, Austria) software or QPath v0.3.0.<br>Clonogenic survival assays were quantified using MATLAB.<br>Immunoflourescence slides were imaged on a Leica SP8 Confocal Microscope. Mitotic phase analysis of the MEL202R625G-DEG and MEL202R625G cell lines was imaged using the Zeiss Axio Observer Z1 Advanced Marianas™ Microscope attached with a CSU-W1 SoRa and quantified by eye.
Data analysis	Statistical analysis was carried out using R 3.5.0 (www.r-project.org) and GraphPad Prism 9.<br>RNA sequencing FASTQ files were aligned to the human genome (hg38) using STAR v2.5.1b with the additional custom parameters '--twopassMode Basic --outSAMstrandField intronMotif --outSAMattributes NH HI AS nM NM XS' with transcript annotations obtained from GENCODE version 22.<br>Differential gene expression analysis was performed using a negative binomial generalised log-linear model (glmQLFit and glmQLFTest) implemented in edgeR v3.34.0. Normalisation factors to correct for variable sequencing depth and composition bias were calculated using the using the Trimmed Mean of M-values (TMM) method (calcNormFactors). Gene Set Enrichment Analysis was performed with FGSEA version 1.4.1 using the c2.cp.reactome gene sets obtained from the Broad Institute with the minimum pathway size set to 10.<br>Quantification of PSI values for the alternative splicing event types (Alternative '5, Alternative '3, Exon skip, Multiple Exon Skip, Intron Retention) was performed with spladder (development version dated: 20180703) under default settings (confidence level = 3). rMATS v4.1.2 under default parameters was used as second method to identify and quantify alternative splicing events. Detection of differential alternative splicing events from both spladder and rMATS between K562 SF3B1WT and SF3B1K700E cells was assessed by performing a differential PSI

analysis using the limma v3.48.3. Sequence motif logos illustrating 30 bp upstream and 3bp downstream of significant alternative 3′ acceptor splice sites were generated using ggseqlogo v0.1.

RNA PolII ChIP-seq data were mapped to the genome using BWA algorithm vv0.7.12 with default settings and hg38 reference genome. Only reads that passed Illumina's purity filter, aligned with no more than 2 mismatches, and mapped uniquely to the genome were used in the subsequent analysis. Peaks were called using the SICER v1.1.

Clonogenic assay NALM-6 and K562 cell lines were imaged without fixation and quantified on MATLAB vR20018b(9.5.0)

For the proteomics data, the raw files were processed with Proteome Discoverer 2.3 (Thermo Fisher) and searched using both SequestHT and Mascot (v2.3 MatrixScience) against UniProt Human Reference Proteome database (January 2018) concatenated with the cRAP contaminate sequences.

Immunofluorescence was quantified using CellProfiler (version 3.1.9). Foci were counted using the "Speckle Counting" pipeline, while phosphor-histone H3, Cajal Body, p21 and nuclear area analysis was performed using the "Cell/particle counting and scoring the percentage of stained objects" pipeline.

For manuscripts utilizing custom algorithms or software that are central to the research but not yet described in published literature, software must be made available to editors and reviewers. We strongly encourage code deposition in a community repository (e.g. GitHub). See the Nature Portfolio guidelines for submitting code & software for further information.

Data

Policy information about availability of data

All manuscripts must include a data availability statement. This statement should provide the following information, where applicable:

- Accession codes, unique identifiers, or web links for publicly available datasets
- A description of any restrictions on data availability
- For clinical datasets or third party data, please ensure that the statement adheres to our policy

The data that support the findings of this study are available in the Supporting Information. The RNA sequencing data have been deposited in NCBI Sequence Read Archive (SRA) under accession number PRJNA849566; ChIP-seq data PRJNA968072 and the mass spectrometry proteomics data have been deposited to the ProteomeXchange Consortium via the PRIDE partner repository with the dataset identifier PXD019046.

SF3B1 mutations were collated from cBioPortal https://www.cbioportal.org/ querying MSK-IMPACT PanCancer Clinical Sequencing cohort and TCGA Pan Cancer Atlas studies. Database access 07/2020.
UniProt Human Reference Proteome database (January 2018) was used as a reference for the Mass-Spectrometry data.

Human research participants

Policy information about studies involving human research participants and Sex and Gender in Research.

Reporting on sex and gender	This is available in the original publication Pratt et al doi:10.1111/bjh.14793
Population characteristics	This is available in the original publication Pratt et al doi:10.1111/bjh.14793
Recruitment	This is available in the original publication Pratt et al doi:10.1111/bjh.14793
Ethics oversight	This is available in the original publication Pratt et al doi:10.1111/bjh.14793

Note that full information on the approval of the study protocol must also be provided in the manuscript.

Field-specific reporting

Please select the one below that is the best fit for your research. If you are not sure, read the appropriate sections before making your selection.

☒ Life sciences ☐ Behavioural & social sciences ☐ Ecological, evolutionary & environmental sciences

For a reference copy of the document with all sections, see nature.com/documents/nr-reporting-summary-flat.pdf

Life sciences study design

All studies must disclose on these points even when the disclosure is negative.

Sample size	Sample sizes for in vivo studies were based on the lowest number of animals required to give a probability of a type I error of 0.05 with a power of 80% assuming a 50% mean difference in drug effect between treatment and control arms in each study. This equated to 12 animals per arm of each study given a take rate of at least 70%.

Data exclusions | No mice were excluded

Replication | Sample sizes were sufficiently powered to enable robust reproducibility. Aside from in vivo experiments, replicate experiments were performed in single, duplicate or triplicate independent biological replicates as stated in the figure legends. All data was reproduced in replicate experiments.

Randomization | Animals were randomised when tumours reached 100mm3. All other experiments were allocated into experimental groups based on treatment (PARPi or control) and/or SF3B1 mutation status (mutant or wild-type).

Blinding | For all in vivo studies, the investigators were blinded to group allocations and dosing was performed by independent lab technicians.

Reporting for specific materials, systems and methods

We require information from authors about some types of materials, experimental systems and methods used in many studies. Here, indicate whether each material, system or method listed is relevant to your study. If you are not sure if a list item applies to your research, read the appropriate section before selecting a response.

Materials & experimental systems

n/a	Involved in the study
☐	☒ Antibodies
☐	☒ Eukaryotic cell lines
☒	☐ Palaeontology and archaeology
☐	☒ Animals and other organisms
☐	☒ Clinical data
☒	☐ Dual use research of concern

Methods

n/a	Involved in the study
☐	☒ ChIP-seq
☐	☒ Flow cytometry
☒	☐ MRI-based neuroimaging

Antibodies

Antibodies used

Table S9. Antibodies and dilutions
Catalogue No. Supplier Antibody Dilution Application in this study Lot Number
MAB3802 Millipore Anti-53BP1 Antibody, clone BP13 1:1000 IF 3524755
ab180955 Abcam Anti-CINP antibody [EPR14446] ab180955 1:1000 WB, IHC GR148706-2
AB87913 Abcam Anti-Coilin antibody [IH10] (ab87913) 100ug 1:1000 IF GR3218582-3
7076S Cell Signalling Technology Anti-mouse IgG, HRP-linked Antibody #7076 1:5000 WB 36
05-636 Millipore Anti-phospho-Histone H2A.X (Ser139) Antibody, clone JBW301 1:1000 IF 3313712
7074S Cell Signalling Technology Anti-rabbit IgG, HRP-linked Antibody #7074 1:5000 WB 30
NA18 Millipore Anti-Replication Protein A (Ab-2) Mouse mAb (RPA34-19) 1:200 IF 3173547
30632S Cell Signalling Technology ATR (phospho Thr1989) antibody 1:1000 WB 1
2360S Cell Signalling Technology Chk1 (2G1D5) Mouse mAb #2360 1:1000 WB 3
2639S Cell Signalling Technology Fibrillarin (C13C3) Rabbit mAb #2639 1:1000 IF 2
ab133741 Abcam Lamin B1 1:1000 WB GR3244890-2
3873S Cell Signalling Technology Monoclonal Anti-α-Tubulin antibody produced in mouse 1:1000 WB 16
2947T Cell Signalling Technology p21 Waf1/Cip1 (12D1) Rabbit mAb #2947 1:1000 WB, IF 11
2344S Cell Signalling Technology Phospho-Chk1 (Ser317) Antibody #2344 1:1000 WB 12
2348S Cell Signalling Technology Phospho-Chk1 (Ser345) (133D3) Rabbit mAb #2348 1:1000 WB 18
2197S Cell Signalling Technology Phospho-Chk2 (Thr68) (C13C1) Rabbit mAb 1:1000 WB 12
53348S Cell Signalling Technology Phospho-Histone H3 (Ser10) (D7N8E) XP® Rabbit mAb #53348 1:1000 IF 1
ab183519 Abcam Recombinant Anti-CINP antibody [EPR14445] - N-terminal 1:1000 WB GR153682-4
ab133534 Abcam Recombinant Anti-Rad51 antibody [EPR4030(3)] 1:1000 IF GR219215-42
2808S Cell Signalling Technology Survivin (71G4B7) Rabbit mAb #2808 1:1000 WB, IF 15
18799S Cell Signalling Technology Vinculin (E1E9V) XP® Rabbit mAb (HRP Conjugate) 1:1000 WB 2
sc53382 Santa Cruz MUS81 (MTA30 2G10/3) mouse mAb monoclonal 1/100 1:1000 IF G0721
NB100-182 Novus FANCD2 Rabbit polyclonal Ab 1/400 1:400 IF S-5
4526S Cell Signalling Technology Phospho-ATM (Ser1981) (10H11.E12) Mouse mAb 1:1000 WB 14
2978S Cell Signalling Technology Cyclin D1 (92G2) Rabbit mAb 1:1000 WB 13
2737S Cell Signalling Technology ATRIP Antbody (Rabbit) 1:1000 WB 2
14793S Cell Signalling Technology DYKDDDDK Tag (D6W5B) Rabbit mAb 1:1000 WB 7
5125S Cell Signalling Technology β-Actin (13E5) Rabbit mAb (HRP Conjugate) 1:5000 WB 6
ab26721 Abcam Anti-RNA polymerase II CTD repeat YSPTSPS antibody - ChIP Grade 1:1000 WB n/a
MABE954 Sigma-Aldrich Anti-phospho RNA Pol II (Ser5), clone 1H4B6 Antibody 1:1000 WB 3512558
MABE953 Sigma-Aldrich Anti-phospho RNA Pol II (Ser2), clone 3E7C7 Antibody 1:1000 WB 3692727
A300-996A Bethyl Laboratories SF3b155/SAP155 Polyclonal Antibody 1:1000 WB 1
WH0000142M1 Sigma-Aldrich Anti-PARP1 Monoclonal Antibody 1:1000 WB KC101-3G4
ab51052 Abcam Recombinant Anti-Hsc70 Rabbit mAb (EP1531Y) 1:1000 WB n/a
39097 Active Motif Anti- RNA Polymerase II (total) Mouse mAb (Clone H48) 20uL 4ug (ChIP-seq) WB 19
347580 BD Biosciences anti- BrdU. Mouse mAb Clone 3D4 (RUO) 1:20 IF 2077345
ab6326 Abcam anti- BrdU. rat mAb 1:400 IF GR3365969-8

Validation

Table S9. Antibodies and dilutions
Catalogue No. Validated in this study Validation by company
MAB3802 WB, ChIP, Flow, Flow-IC, IB, ICC/IF, IHC, IHC-Fr, IHC-P, IP, ISH, KD, KO
ab180955 KD WB, IP
AB87913 WB, ICC, IP, IHC-P, Flow, KO
7076S WB
05-636 WB, IF, ICC, ChIP
7074S WB
NA18 IF, IP
30632S WB
2360S WB, KD
2639S WB, IF
ab133741 WB, IP, ICC/IF, IHC-P, IP, KO
3873S WB, IHC-P, ICC/IF, Flow
2947T WB, IP, IHC, IF, Flow, KO
2344S WB
2348S WB, IF, Flow
2197S WB, IP, IHC, Flow
53348S WB, IP, ICC/IF, ChIP, Flow
ab183519 KD WB, ICC/IF, IP
ab133534 WB, IHC-P, ICC/IF, IP, Flow
2808S WB, IP, IHC-P, ICC/IF, Flow, KD
18799S WB
sc53382 WB, IP
NB100-182 WB, ChIP, Flow, IB, ICC/IF, IHC, IHC-P, IP, KD, KO
4526S WB
2978S WB, IHC-P
2737S WB, IF, IP
14793S WB, IP, IHC-P, ICC/IF, Flow, ChIP
5125S WB
ab26721 WB, IHC-P, IP, ICC/IF, ChIP
MABE954 WB, ChIP-Seq, ICC, ELISA & ChIP
MABE953 WB, ICC, ELISA, ChIP
A300-996A WB, IP
WH0000142M1 KO WB, ELISA, IF
ab51052 IP, Flow-IC, WB, IHC-P, ICC/IF, KO
39097 WB, ChIP, ChIP-Seq
347580 Flow-IC
ab6326 ICC/IF, IHC-P, Flow-IC

Key
WB Western Blot
ChIP Chromatin Immunoprecipitation
Flow Flow cytometry
Flow-IC Flow intracellular
IB Immunobloting
ICC/IF Immunocytochemistry/Immunonblotting
IHC Immunohistochemistry
IHC-Fr Immunohistochemistry-Frozen
IHC-P Immunohistochemistry-Paraffin
IP Immunoprecipitation
ISH In situ hybridisation
KD Knock down validated
KO Knock out validated

Eukaryotic cell lines

Policy information about cell lines and Sex and Gender in Research

Cell line source(s)

All cell lines used in the study are derived from human.
RRID:CVCL_0004. K562 - Female parental SF3B1WT, control edited synonymous mutated SF3B1K700K and mutant SF3B1K700E and SF3B1K666N; and RRID:CVCL_0092. NALM-6 - Male-parental SF3B1WT, control edited synonymous mutated SF3B1K700K and mutated SF3B1H662Q, SF3B1K700E, SF3B1K666N engineered isogenic cell lines were obtained from Horizon Discovery.
RRID:CVCL_C301. MEL202 Female parental cell line was provided by the originator Bruce Kasander Schepens Eye Research Institute; Boston; USA
RRID:CVCL_4D13. MP46 Female patient derived xenograft cell line was provided by the originators Fariba Nemati and Marc Henri-Stern (Institute Curie, France)
RRID:CVCL_C302. MEL270 Male cell line was provided by the originator Bruce Kasander Schepens Eye Research Institute; Boston; USA

	RRID:CVCL_4D12. MP41 Female patient derived xenograft cell line was provided by the originators Fariba Nemati and Marc Henri-Stern (Institute Curie, France)
Authentication	All cell lines were authenticated using STR profiling with the Geneprint10 Kit (Promega) and were sequenced to check the retention of engineered alterations during culture
Mycoplasma contamination	All cell lines were tested monthly to confirm no mycoplasma infection using the MycoalertTM ®Mycoplasma Detection Kit as per manufacturer's instructions. All cell lines used in the study tested negative for mycoplasma infection.
Commonly misidentified lines (See ICLAC register)	No mis-identified lines were used in this study

Animals and other research organisms

Policy information about studies involving animals; ARRIVE guidelines recommended for reporting animal research, and Sex and Gender in Research

Laboratory animals	7-8 week old female CB-17 (NOD.CB17-Prkdcscid/J) , NSG-Nude (NOD.Cg-Foxn1em1Dvs Prkdcscid Il2rgtm1Wjl/J) and NOD-SCID (NOD.Cg-Prkdcscid/J) immuno-compromised mice were purchased from the Jackson Laboratory. All animals were maintained at 24-26°C ambient temperature with 55% humidity. Mice were subject to 12 hour dark-light cycles.
Wild animals	This study did not involve wild animals.
Reporting on sex	Female mice were used in this study
Field-collected samples	This study did not involve samples collected form the field.
Ethics oversight	The in vivo studies carried out at The Institute of Cancer Research were performed to ARRIVE guidelines and regulations as described in the UK Animals Scientific Procedures Act 1986 and according to the UK Home Office projected licences held by CJL and approved by the ethics board at The Institute of Cancer Research (maximum tumour size 15mm diameter). Additional in vivo studies were performed to local regulatory guidelines at Institut Curie (MP41 and MEL202R625G-DEG) (CEEA-IC #118, Authorization APAFiS #25870-2020060410487032-v1 given by National Authority, maximal tumour volume 2500mm3) and Crown Biosciences USA (PDX11310) (maximum tumour size 2000mm3). The maximal tumour size was not exceeded. Patients that provided samples from which PDX were generated were appropriately and fully consented.

Note that full information on the approval of the study protocol must also be provided in the manuscript.

Clinical data

Policy information about clinical studies

All manuscripts should comply with the ICMJE guidelines for publication of clinical research and a completed CONSORT checklist must be included with all submissions.

Clinical trial registration	The trial is registered with ISRCTN registry (ISRCTN34386131)
Study protocol	This is available in the original publication Pratt et al doi:10.1111/bjh.14793
Data collection	This is available in the original publication Pratt et al doi:10.1111/bjh.14793
Outcomes	This is available in the original publication Pratt et al doi:10.1111/bjh.14793

ChIP-seq

Data deposition

☒ Confirm that both raw and final processed data have been deposited in a public database such as GEO.

☒ Confirm that you have deposited or provided access to graph files (e.g. BED files) for the called peaks.

Data access links *May remain private before publication.*	*For "Initial submission" or "Revised version" documents, provide reviewer access links. For your "Final submission" document, provide a link to the deposited data.*
Files in database submission	SRR24460485 PRJNA968071 SAMN34896536 Pooled_Input Pooled_Input<br>SRR24460487 PRJNA968071 SAMN34896534 K700K-WT_Pol2 K700K-WT_Pol2<br>SRR24460486 PRJNA968071 SAMN34896535 K700E-MUT_Pol2 K700E-MUT_Pol2<br>SRR24460488 PRJNA968071 SAMN34896533 Parental_Pol2 Parental_Pol2
Genome browser session (e.g. UCSC)	No longer applicable

Methodology

Replicates	Experiments were performed as a single replicate
Sequencing depth	Single end sequencing 75bp read length.<br>Total number of reads: K562WT- 35,559,728; K562K700K-39,011,608; K562K700E-37,079,970<br>Total mapped(aligned reads): K562WT- 32,001,086; K562K700K-34,594,764; K562K700E-34,072,899<br>Uniquely mapped reads: K562WT- 28,995,696; K562K700K-31,119,159; K562K700E-30,724,511
Antibodies	39097 Active Motif Anti- RNA Polymerase II (total) Mouse mAb 4ug (20uL).
Peak calling parameters	RNA Pol2-enriched regions were identified using the SICER algorithm v. 1.1 at a cutoff of FDR 1E-10 and a max gap parameter of 600 bp.
Data quality	Peaks that were on the ENCODE blacklist of known false ChIP-Seq peaks were removed. Signal maps and peak locations were used as input data to Active Motifs proprietary analysis program, which creates Excel tables containing detailed information on sample comparison, peak metrics, peak locations and gene annotations.<br><br>Filtered peaks: K562WT- 22,534; K562K700K-20,278; K562K700E-20,262
Software	BWA (v0.7.12) genome alignment<br>SICER (V1.1) (peak calling)<br>bcl2fastq2 (v2.20) (processing of Illumina base-call data and demultiplexing)<br>Samtools (v0.1.19) (processing of BAM files)<br>BEDtools (v2.25.0) (processing of BED files)<br>wigToBigWig (v4) (generation of bigWIG files)

Flow Cytometry

Plots

Confirm that:

☒ The axis labels state the marker and fluorochrome used (e.g. CD4-FITC).

☒ The axis scales are clearly visible. Include numbers along axes only for bottom left plot of group (a 'group' is an analysis of identical markers).

☒ All plots are contour plots with outliers or pseudocolor plots.

☒ A numerical value for number of cells or percentage (with statistics) is provided.

Methodology

Sample preparation	Cell-cycle analysis was undertaken using propidium iodide (PI) (Abcam, ab14083) and analysed on BD LSRII cell analyser. Trypsinised cells were washed twice in PBS before fixation through the dropwise addition of 70% ethanol and allowed to fix for 30 min at 4°C. Cell pellets were washed twice with PBS at 850 g and then treated with 50 ul of 100 ug/ml RNase. Finally, 200 ul of 50 ug/ml PI was used to resuspend the cell pellet ready for analysis.
Instrument	All samples were processed on the BD LSRII cell analyser
Software	FlowJO (BD biosciences) analysis software.
Cell population abundance	No sorting was performed.
Gating strategy	Forward and side scatters were set to identify single cells and doublets were excluded. Gates were then automatically set and percentages derived by use of FlowJO (BD biosciences) analysis software.

☒ Tick this box to confirm that a figure exemplifying the gating strategy is provided in the Supplementary Information.
