# Supplementary material for: SF3B1 hotspot mutations confer sensitivity to PARP inhibition by eliciting a defective replication stress response
Source: Nat Genet. 2023 Jul 31;55(8):1311–23. doi: 10.1038/s41588-023-01460-5 (PMC10412459; doi:10.1038/s41588-023-01460-5)

Source Data- Uncropped scans with size markers

MEL202^{R625G} labelled as C37 in some blots

MEL202^{R625G} labelled as MEL202 in some blots

Extended Data Fig. 1f

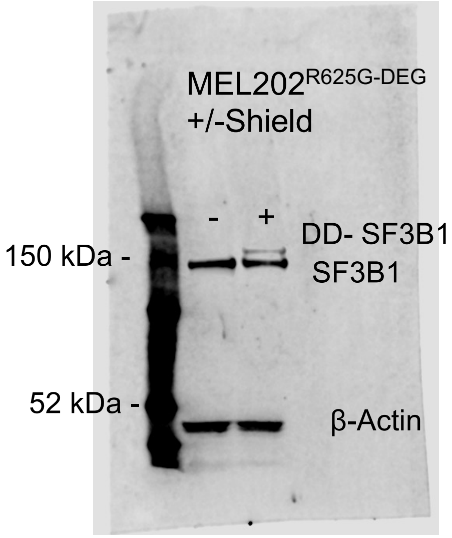

Extended Data Fig. 1j

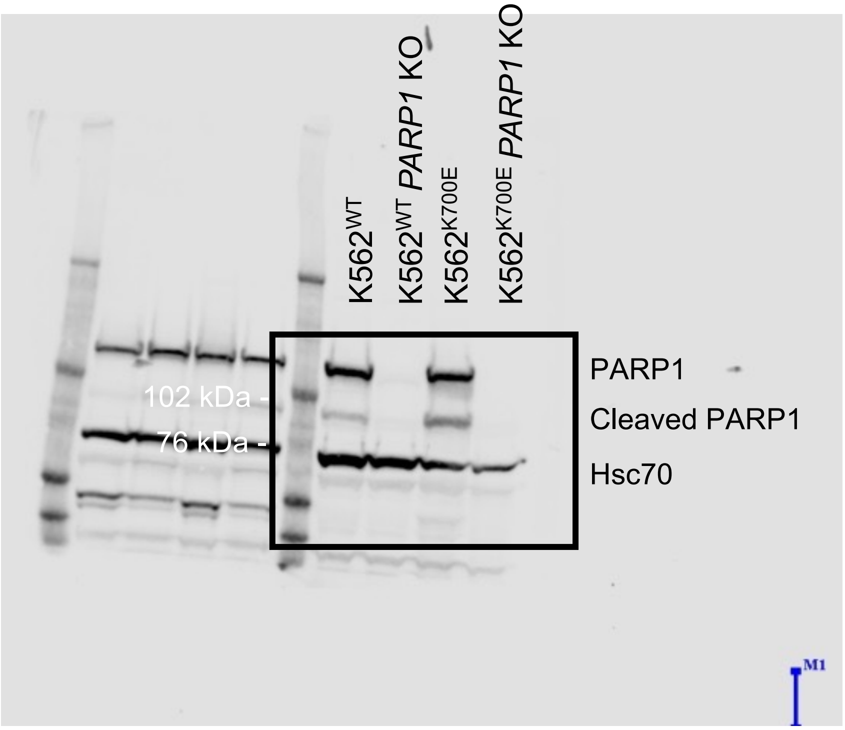

Extended Data Fig. 2e

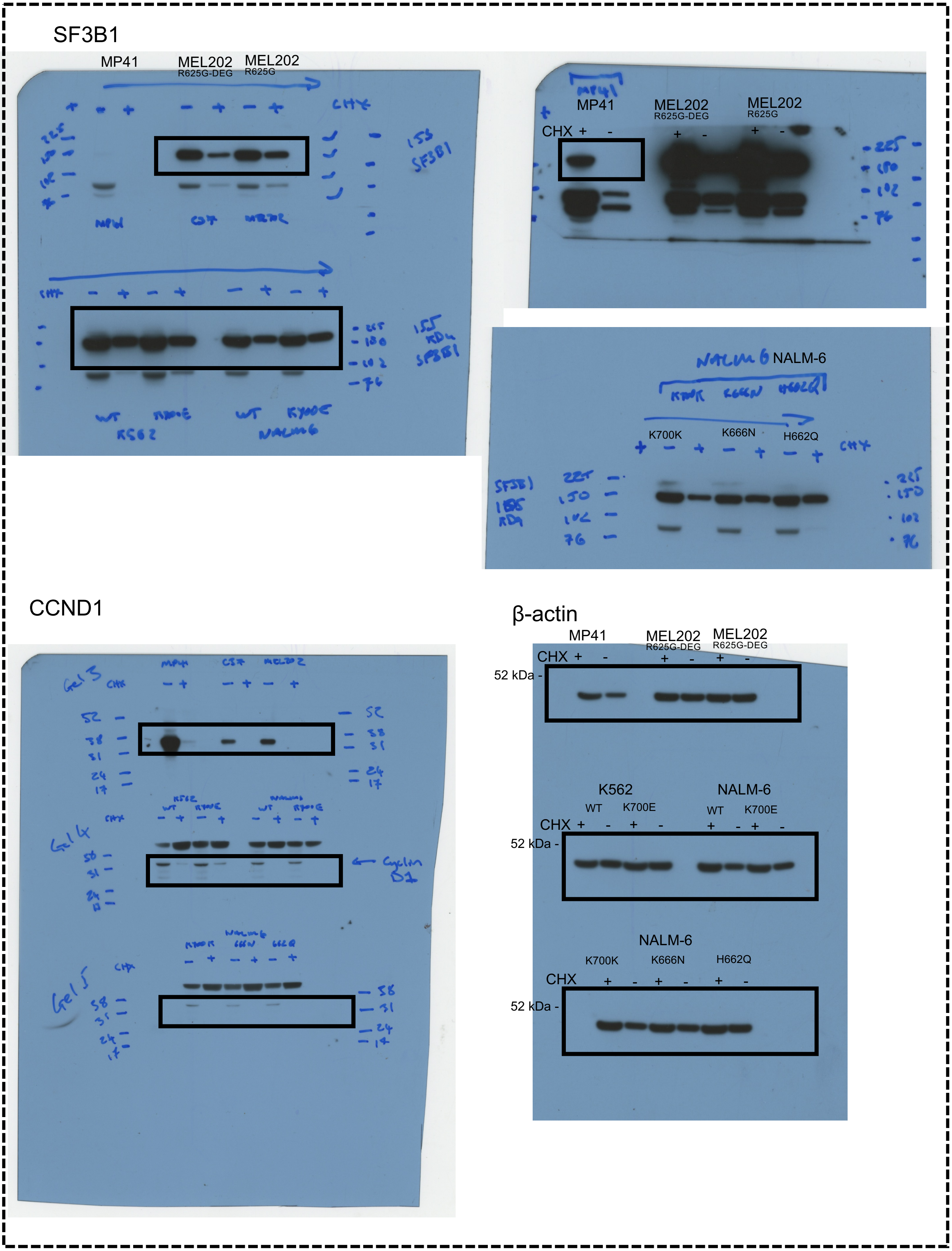

Extended Data Fig. 3e

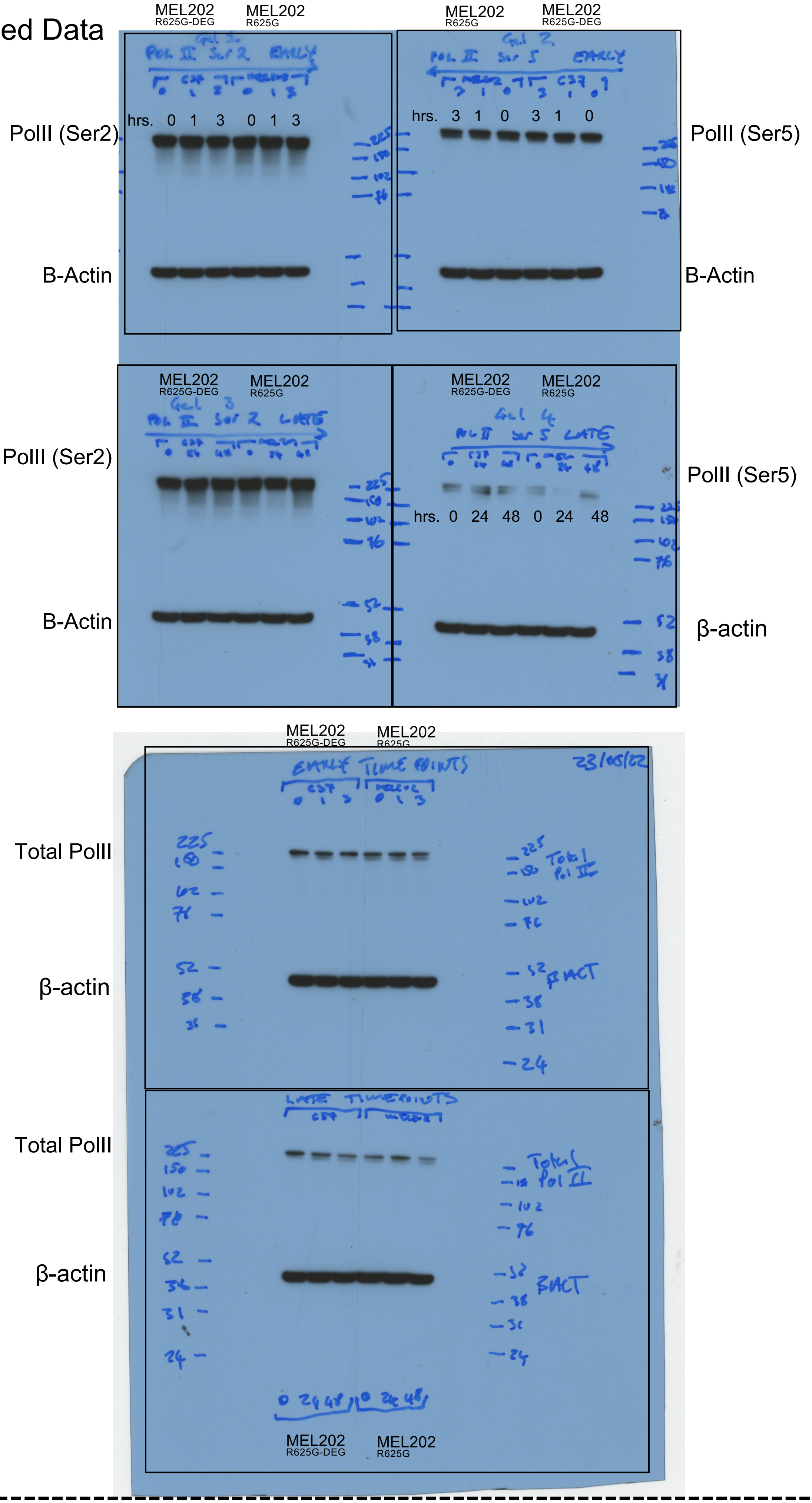

Figure 2f

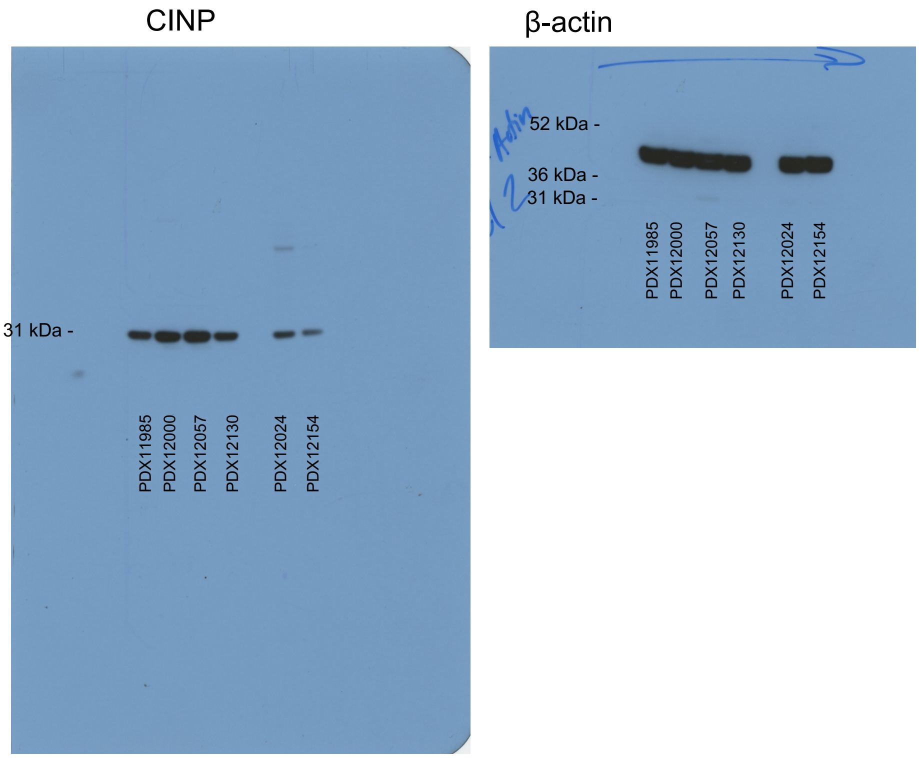

Figure 2h

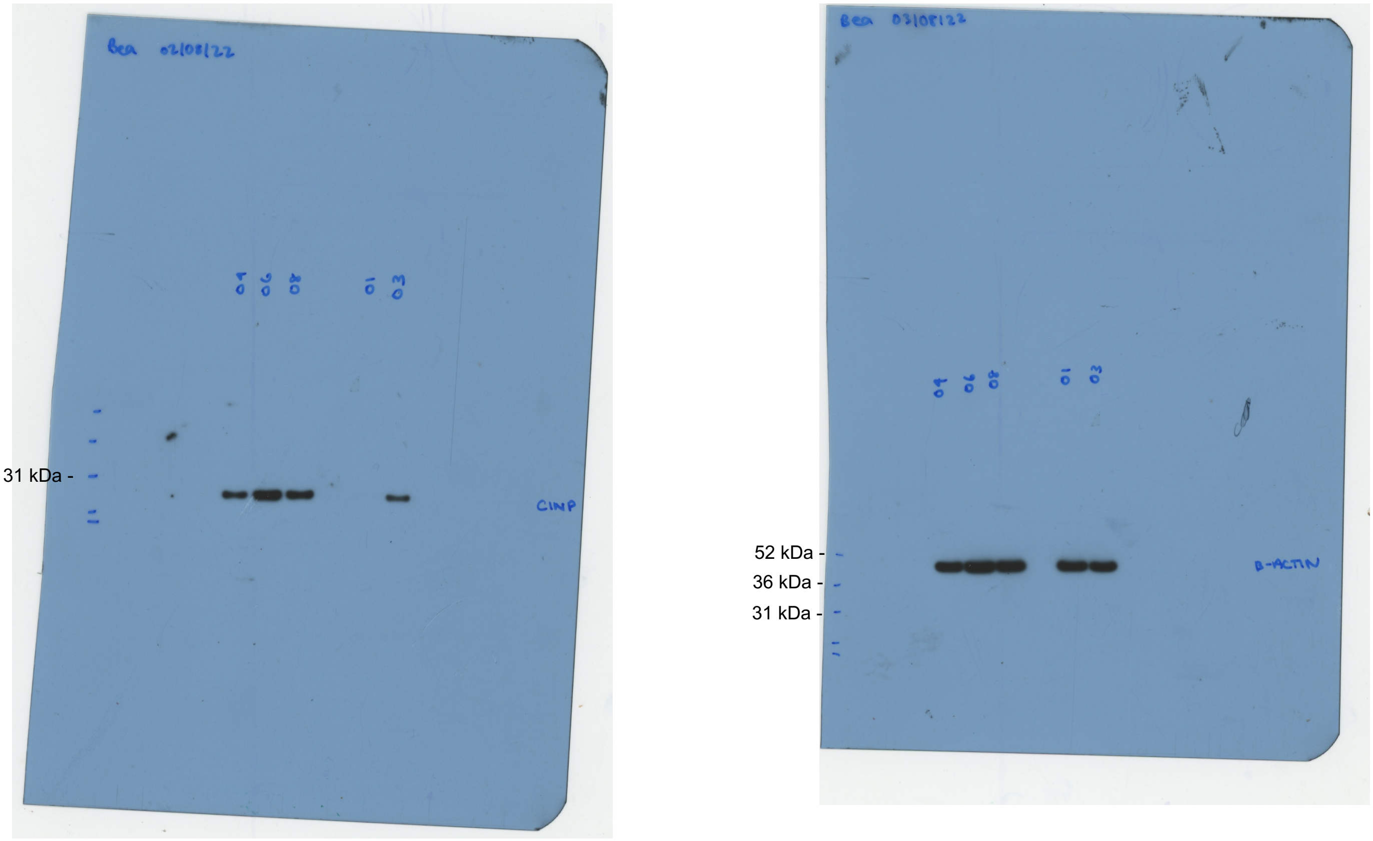

Extended Data Fig. 4b

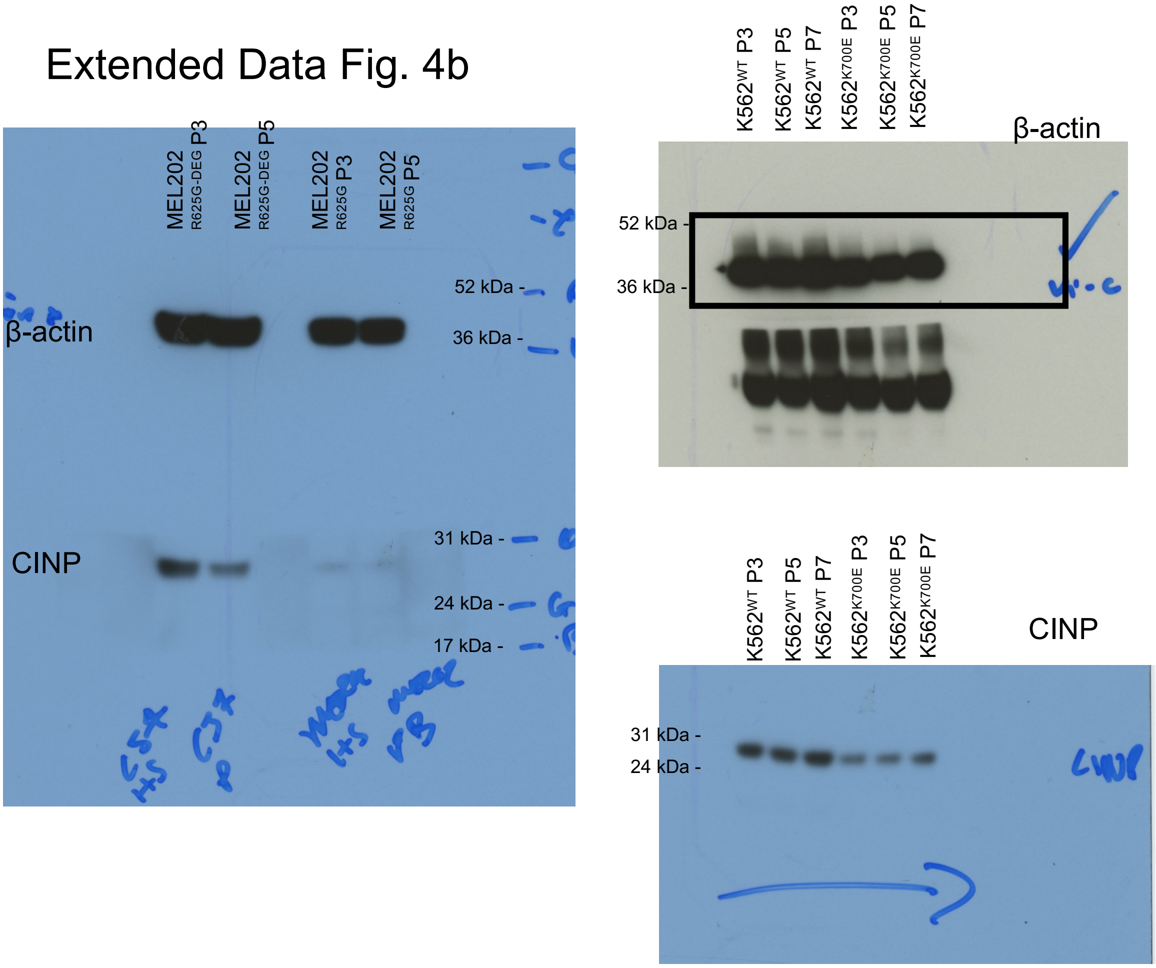

Extended Data Fig. 4b

CINP MP41

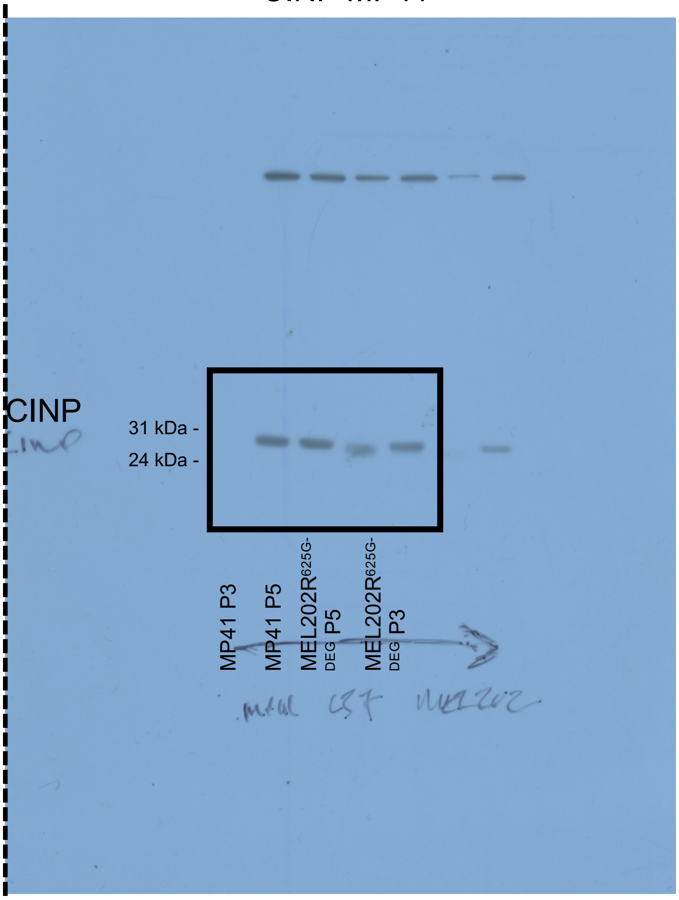

β-actin

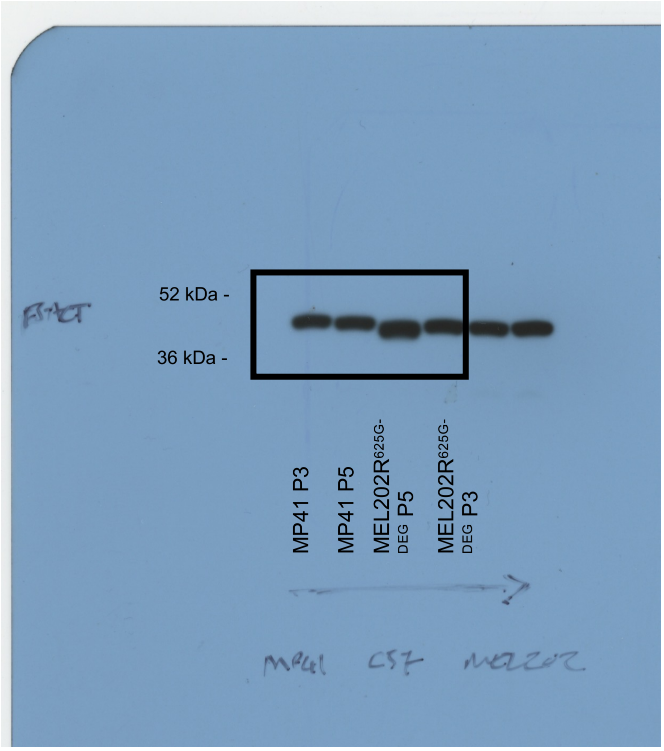

CINP NALM-6 series exposure 1

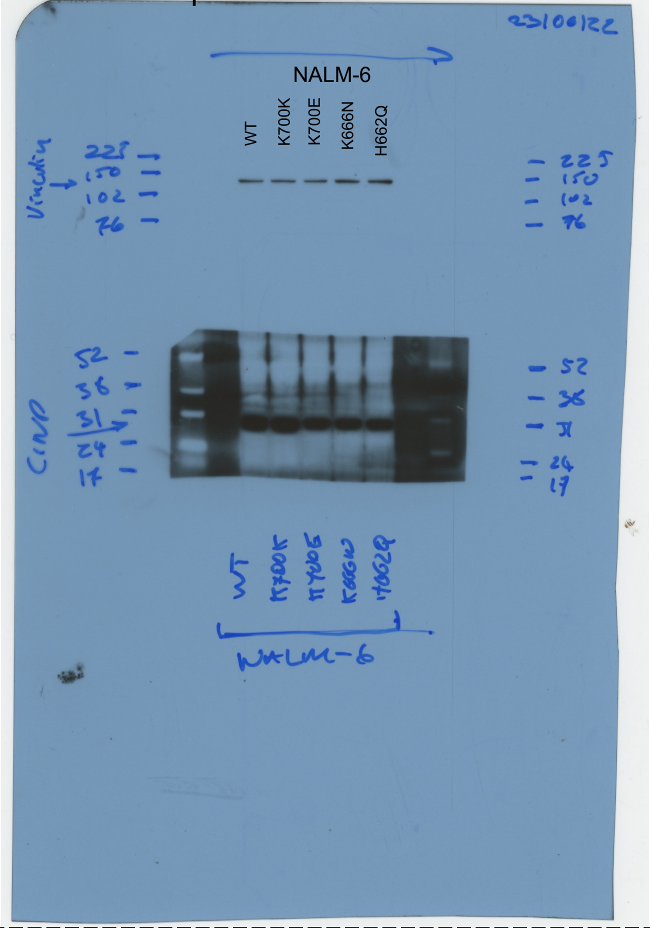

vinculin NALM-6 series exposure used

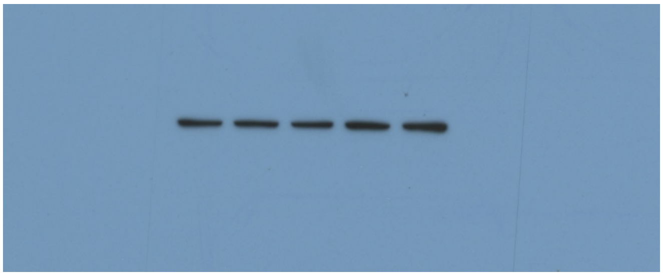

CINP NALM-6 series exposure used

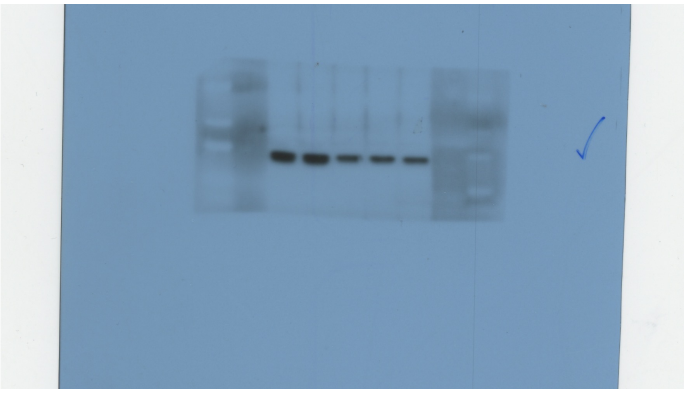

Extended Data Fig. 4c

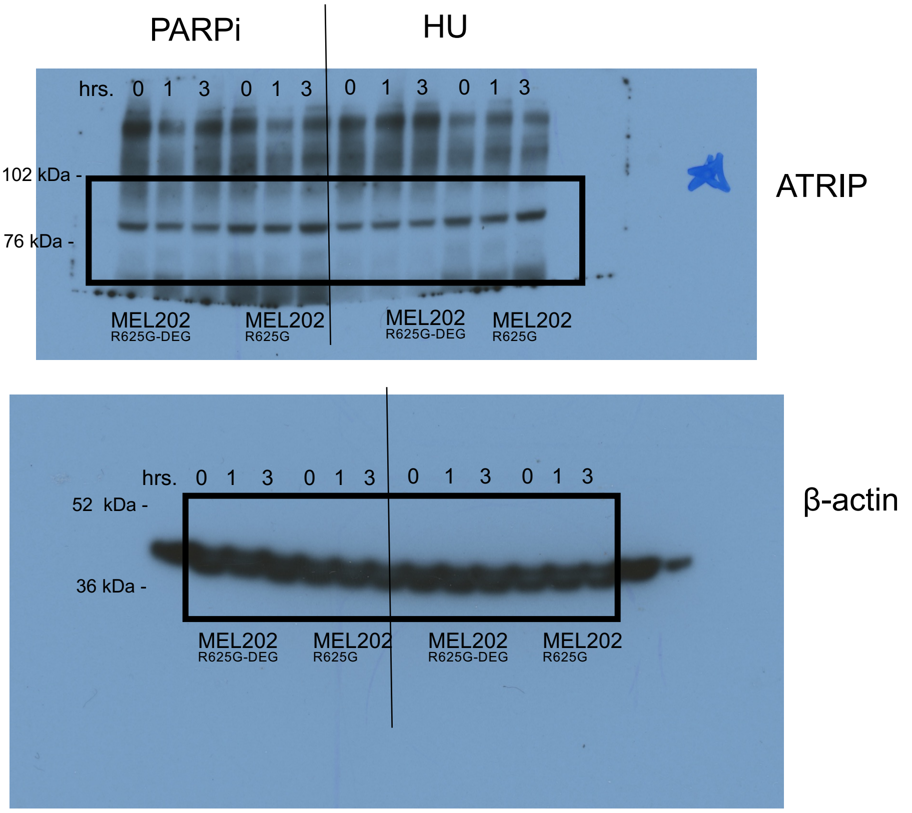

Extended Data Fig. 4d

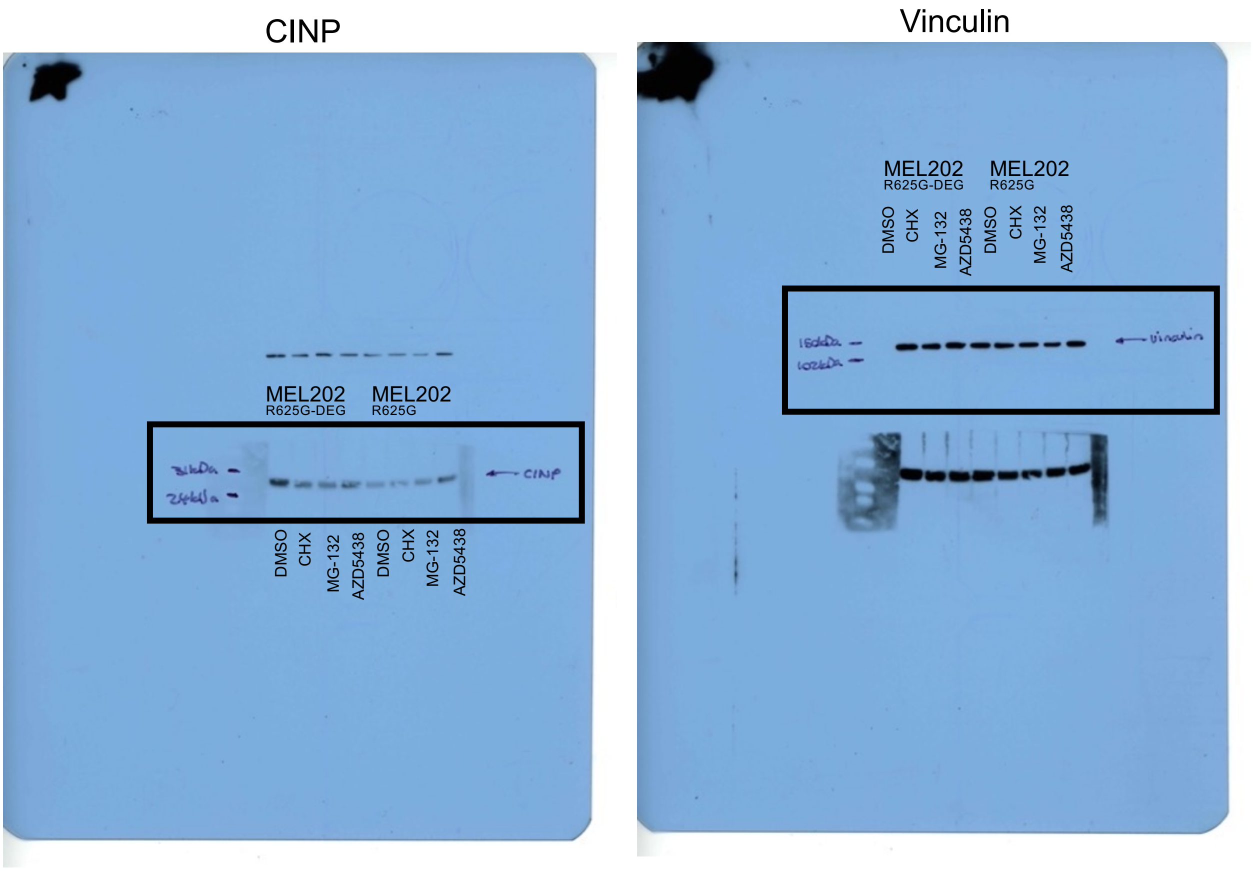

Figure 3e

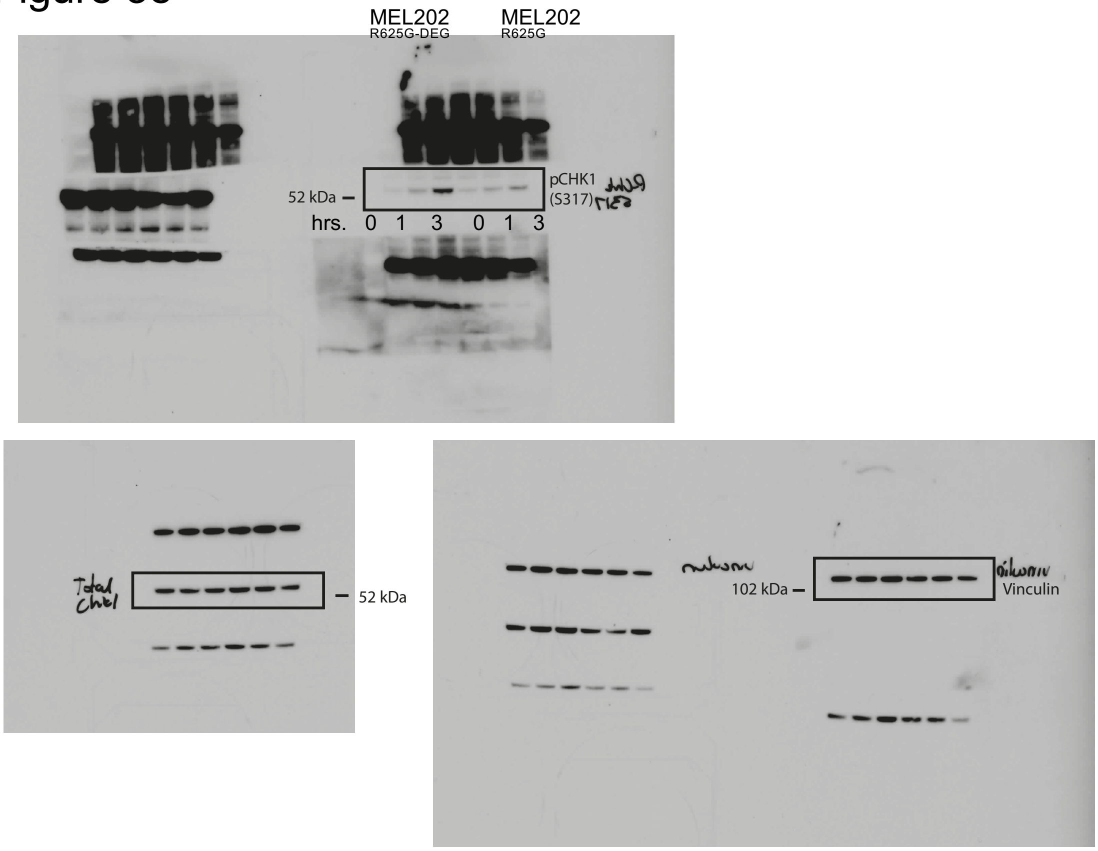

Figure 4a

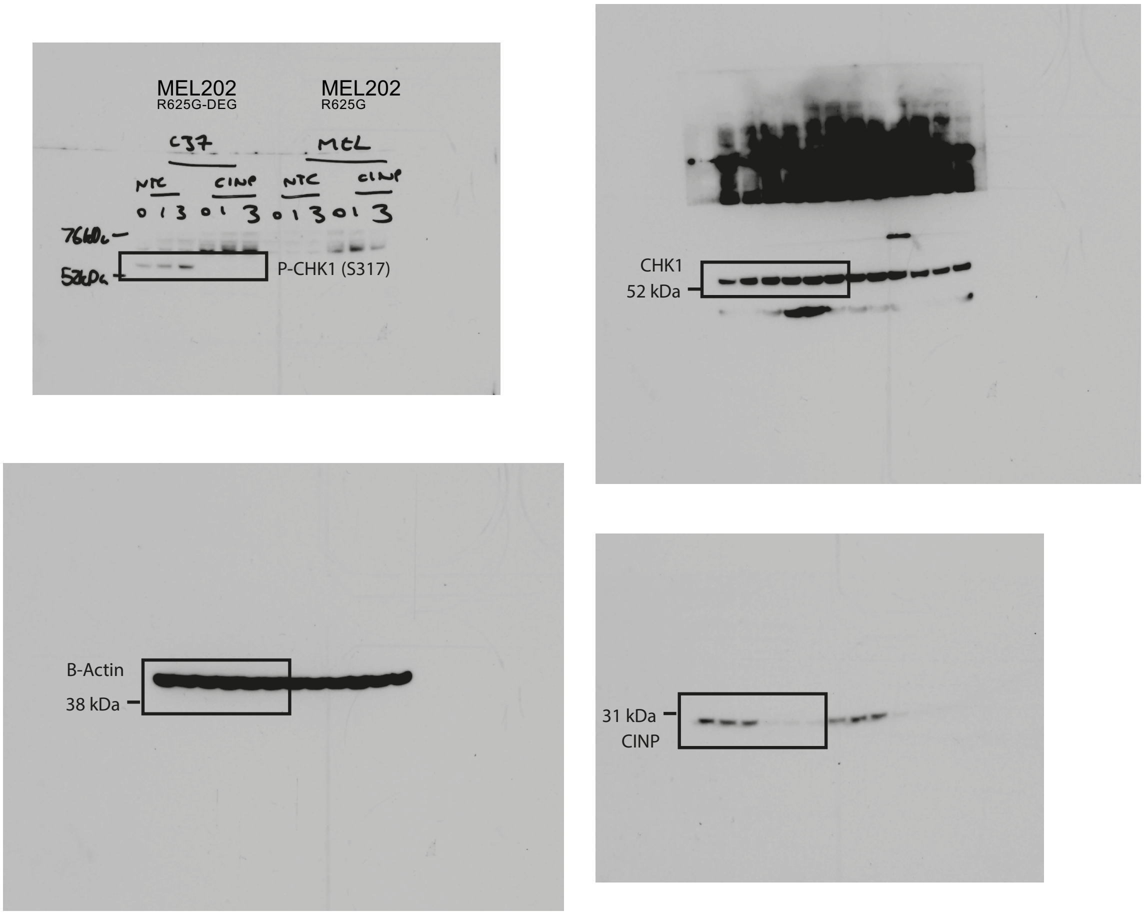

Figure 4c

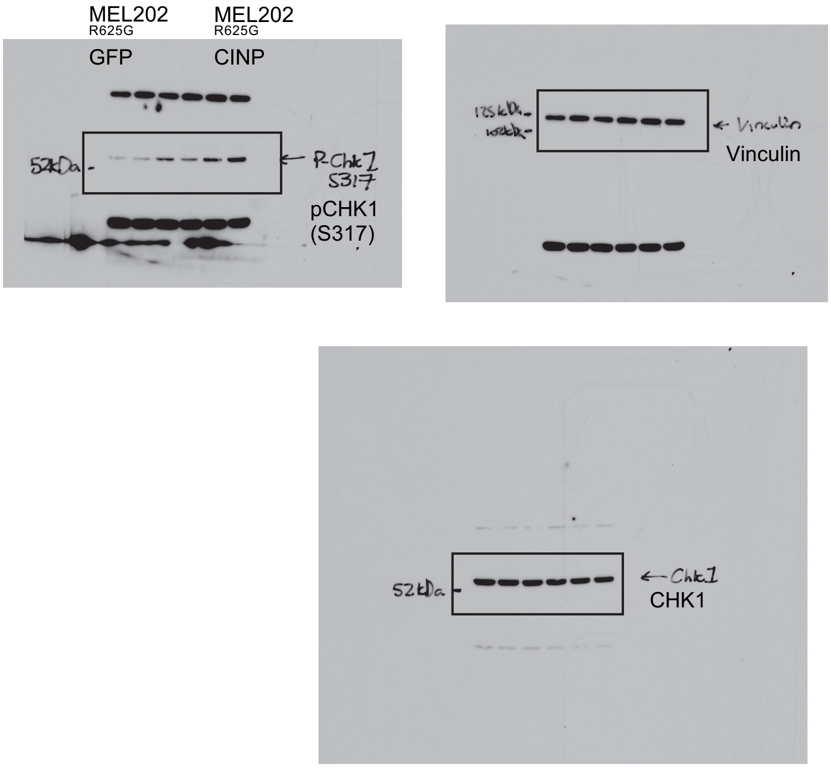
MEL202
R625G
GFP
MEL202
R625G
CINP
pCHK1
(S317)
Vinculin
CHK1

Extended Data Fig. 5di

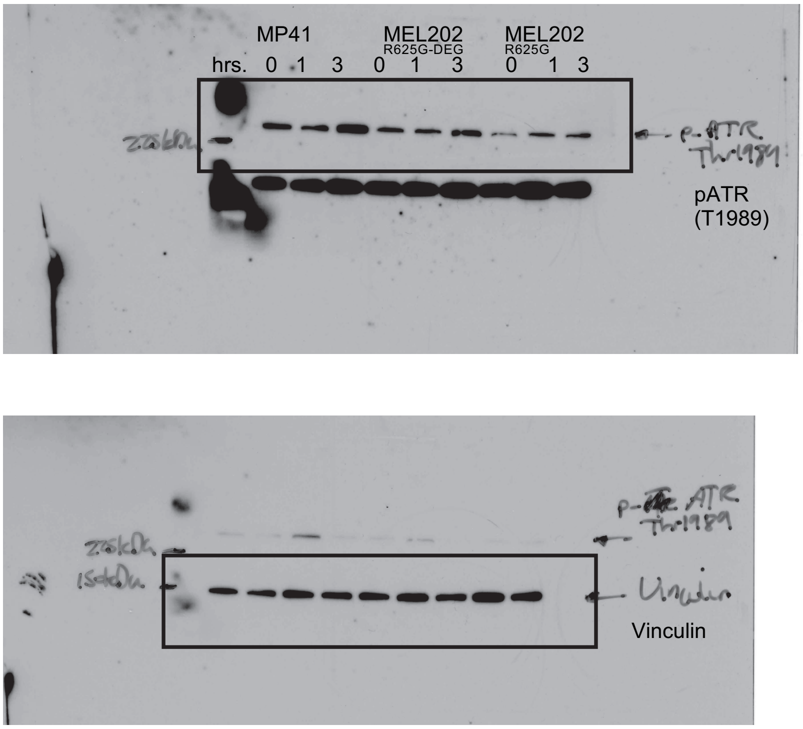

Extended Data Fig. 5dii

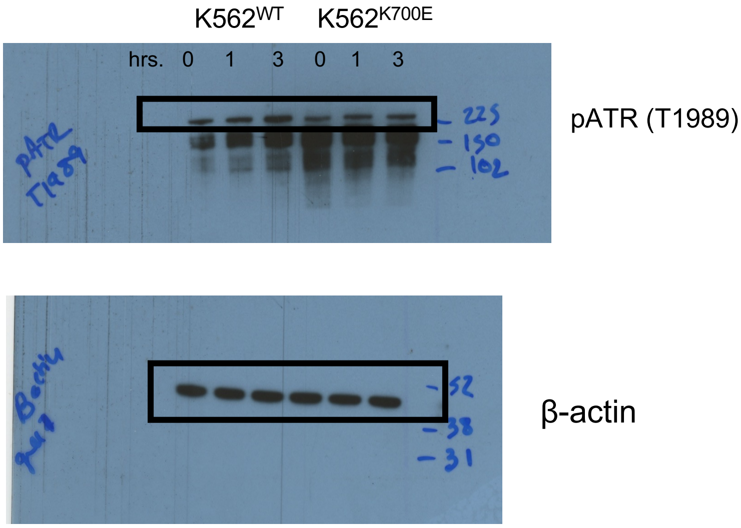

Extended Data Fig. 4e

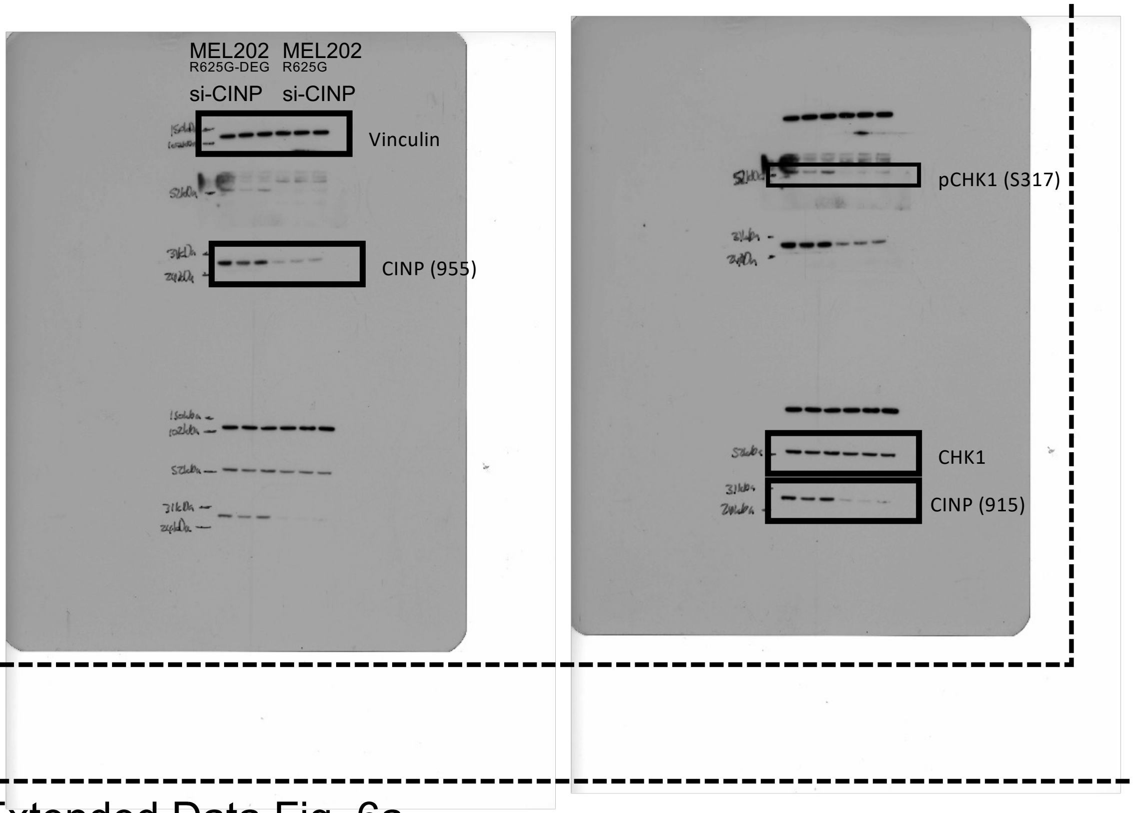

Extended Data Fig. 6a

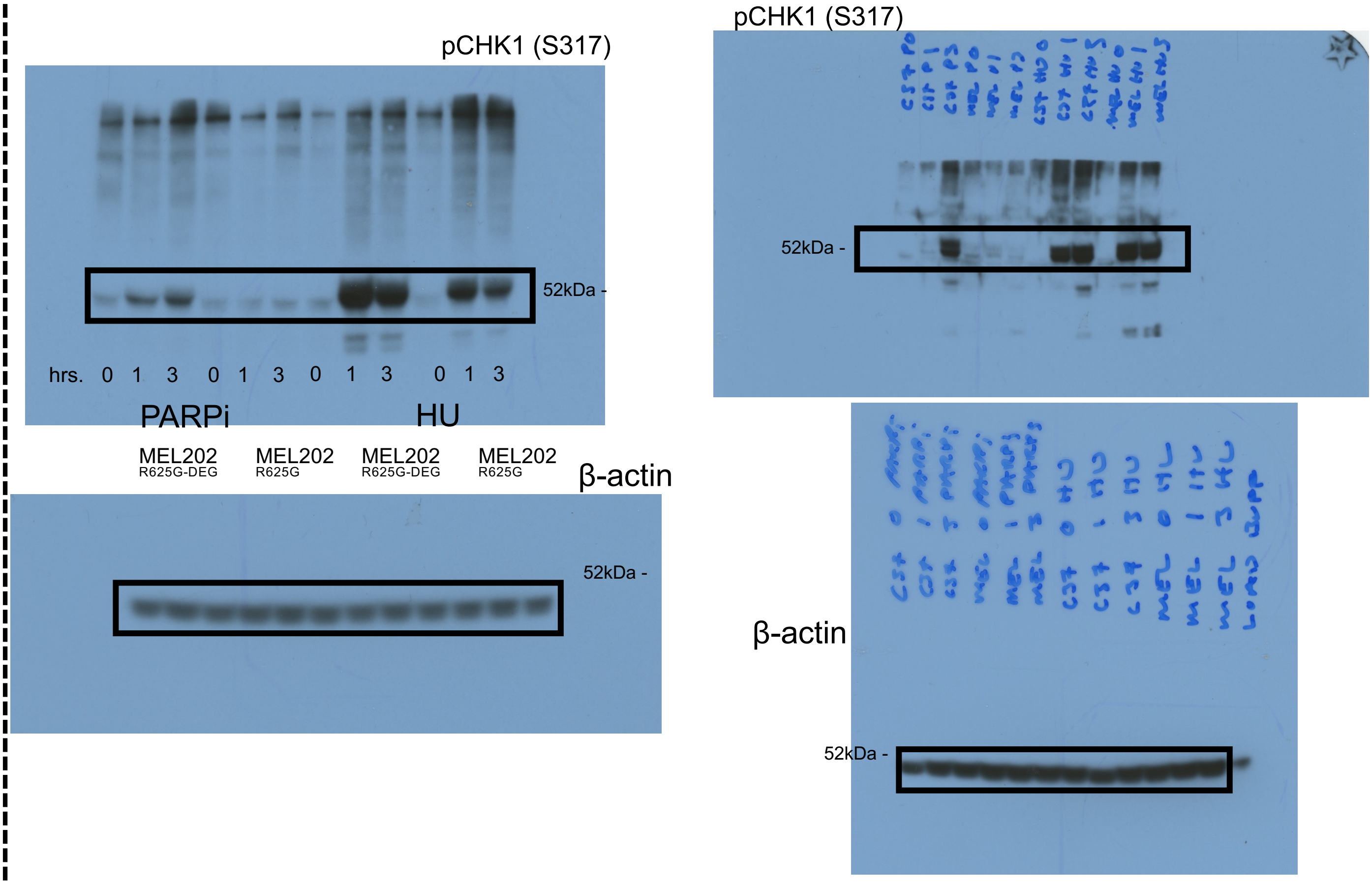

Extended Data Fig. 6b

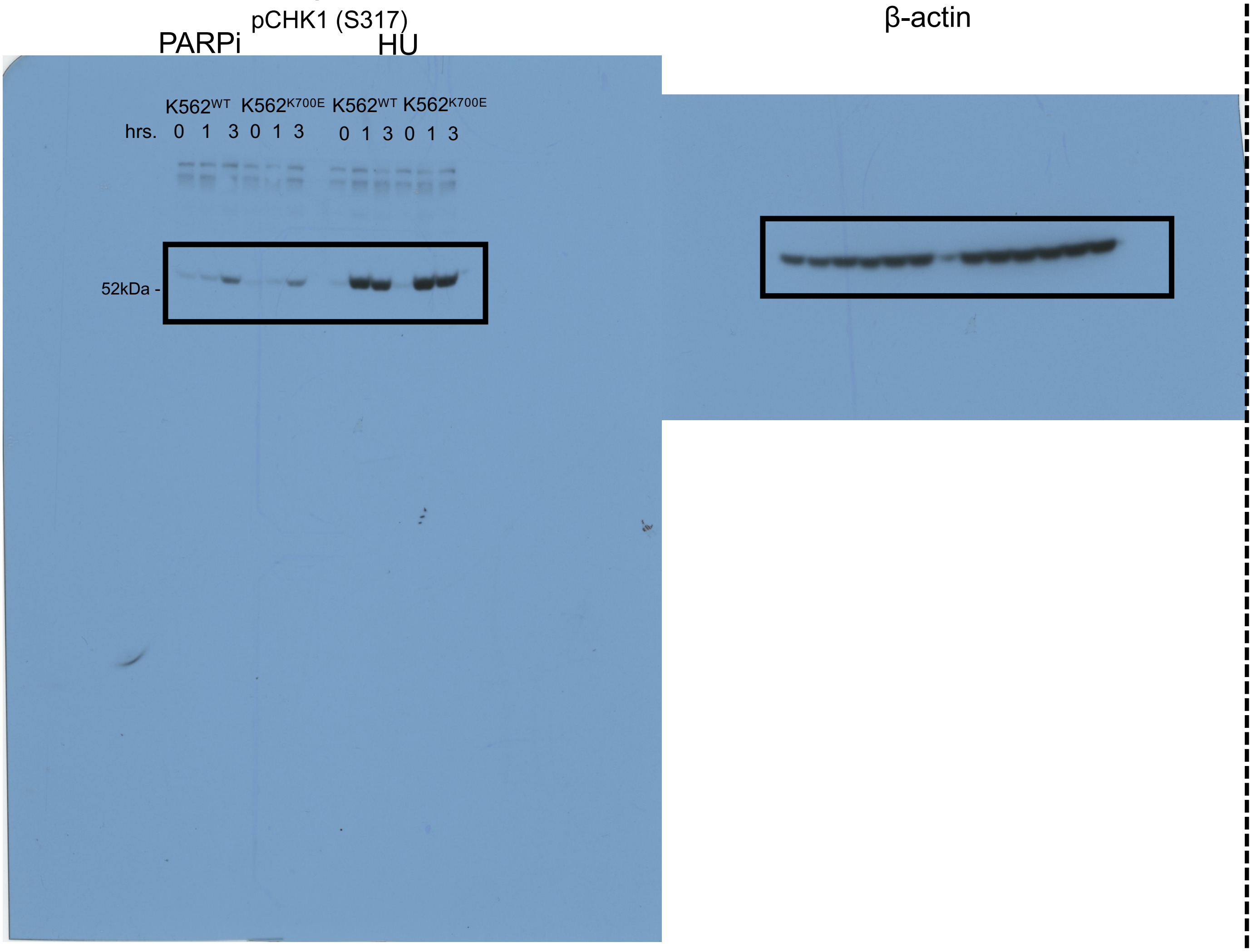

Extended Data Fig. 8d-e

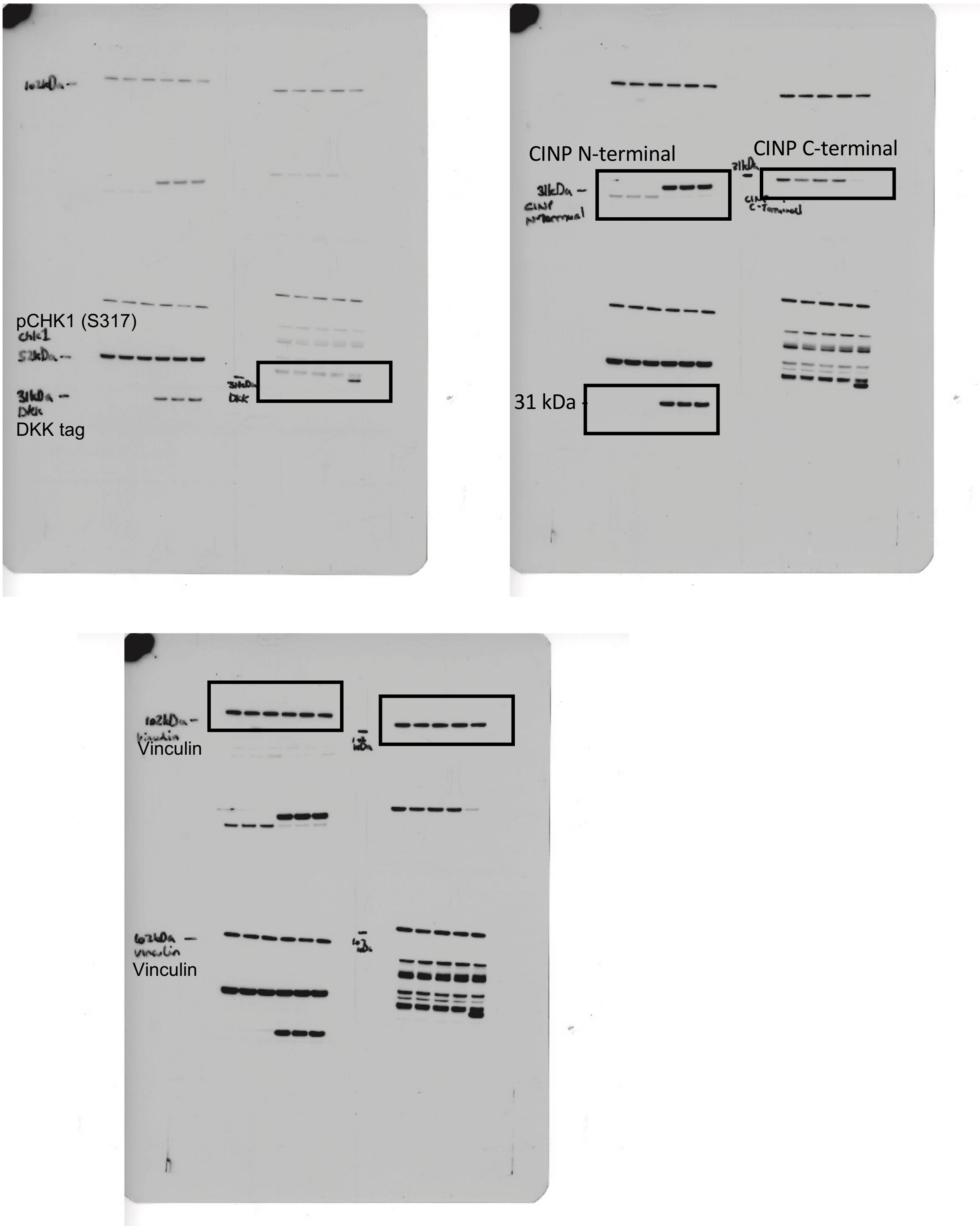

Extended Data Fig. 8h

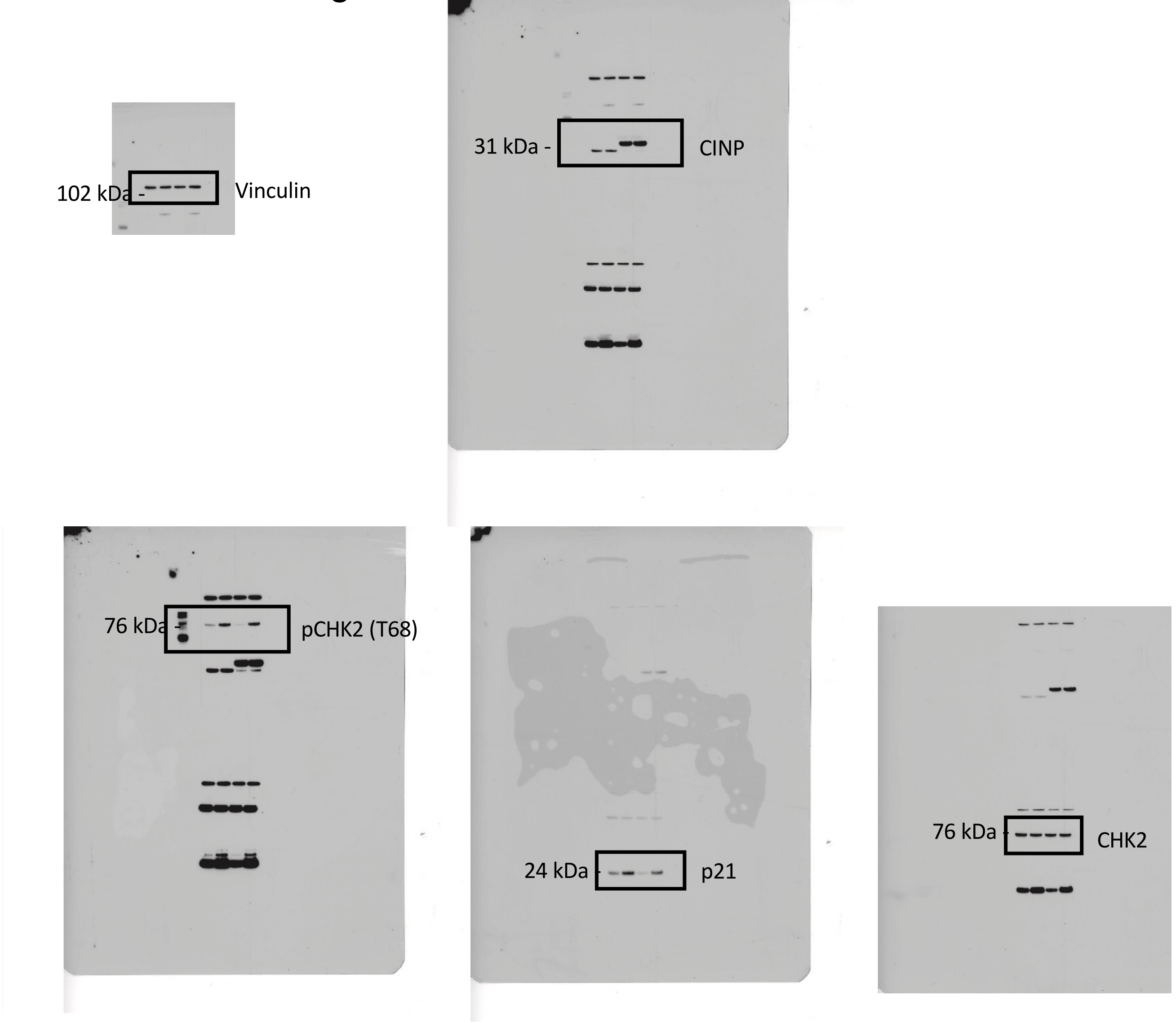

Figure 5b

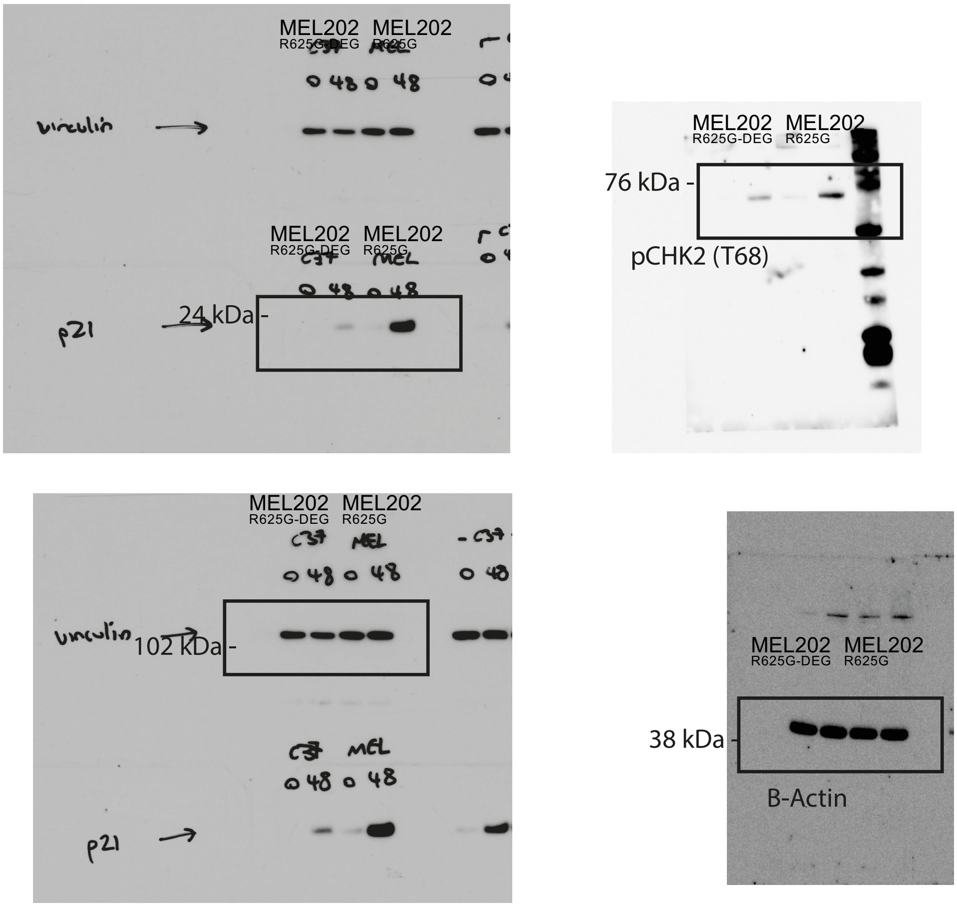
MEL202
R625G-DEG
MEL202
R625G
vinculin
MEL202
R625G-DEG
MEL202
R625G
C37
MEL
0 48 0 48
24 kDa -
p21
MEL202
R625G-DEG
MEL202
R625G
76 kDa -
pCHK2 (T68)
MEL202
R625G-DEG
MEL202
R625G
C37
MEL
0 48 0 48
vinculin
102 kDa -
C37
MEL
0 48 0 48
p21
MEL202
R625G-DEG
MEL202
R625G
38 kDa -
B-Actin

Figure 5d

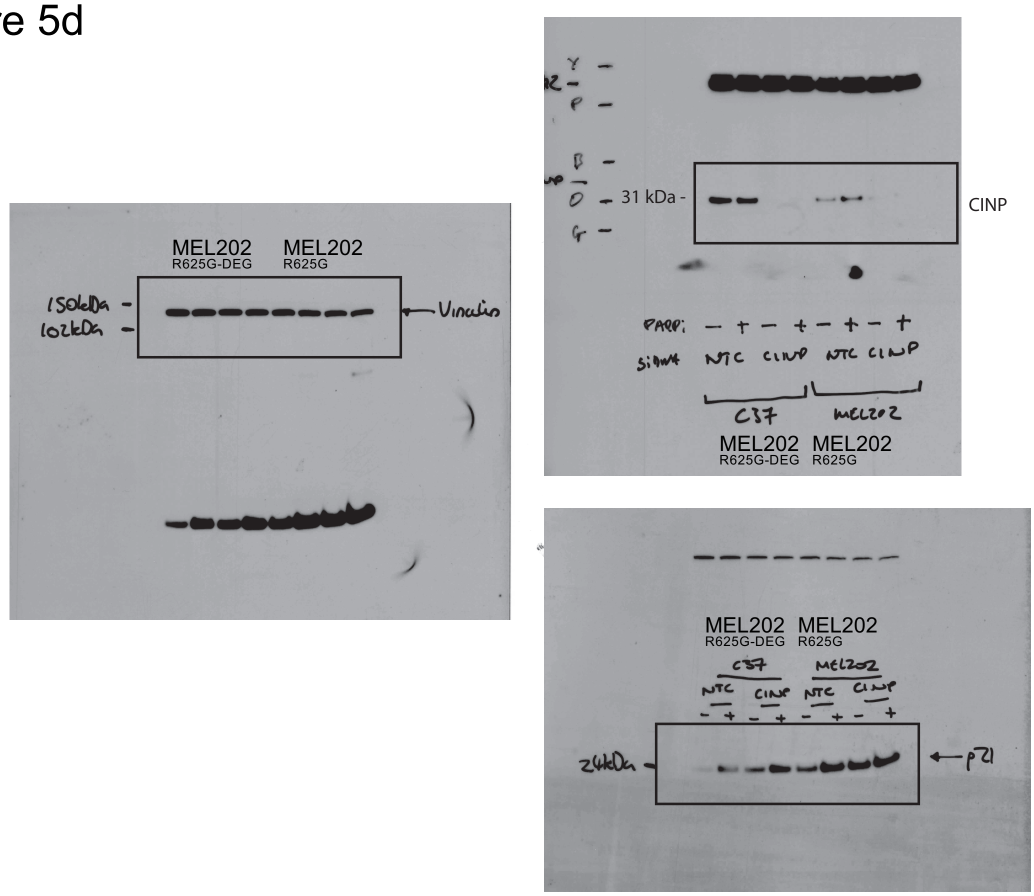
MEL202
R625G-DEG
MEL202
R625G
150kDa
102kDa
Vinculin
31 kDa -
CINP
PARPi
siRNA
NTC
CINP
NTC
CINP
C37
MEL202
MEL202
R625G-DEG
MEL202
R625G
MEL202
R625G-DEG
MEL202
R625G
C37
MEL202
NTC
CINP
NTC
CINP
24kDa
p21

Figure 5e

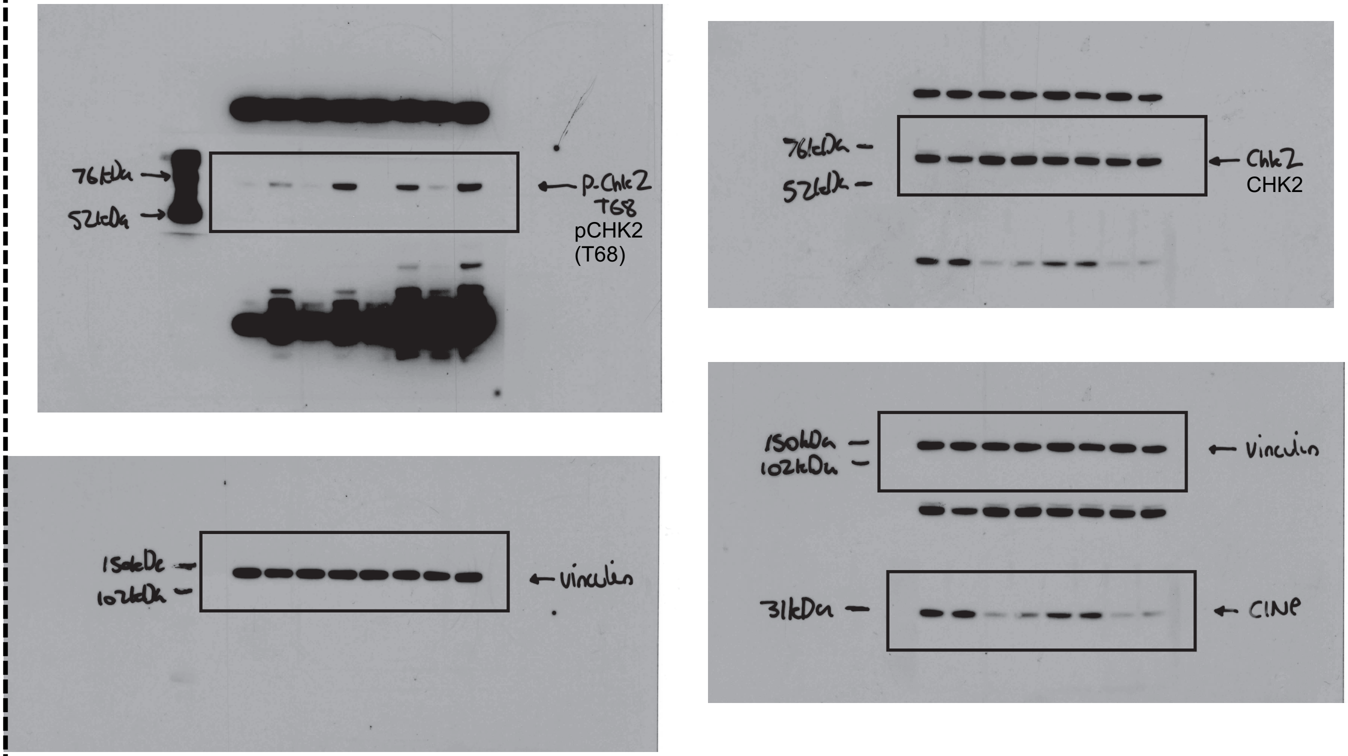

Figure 5g

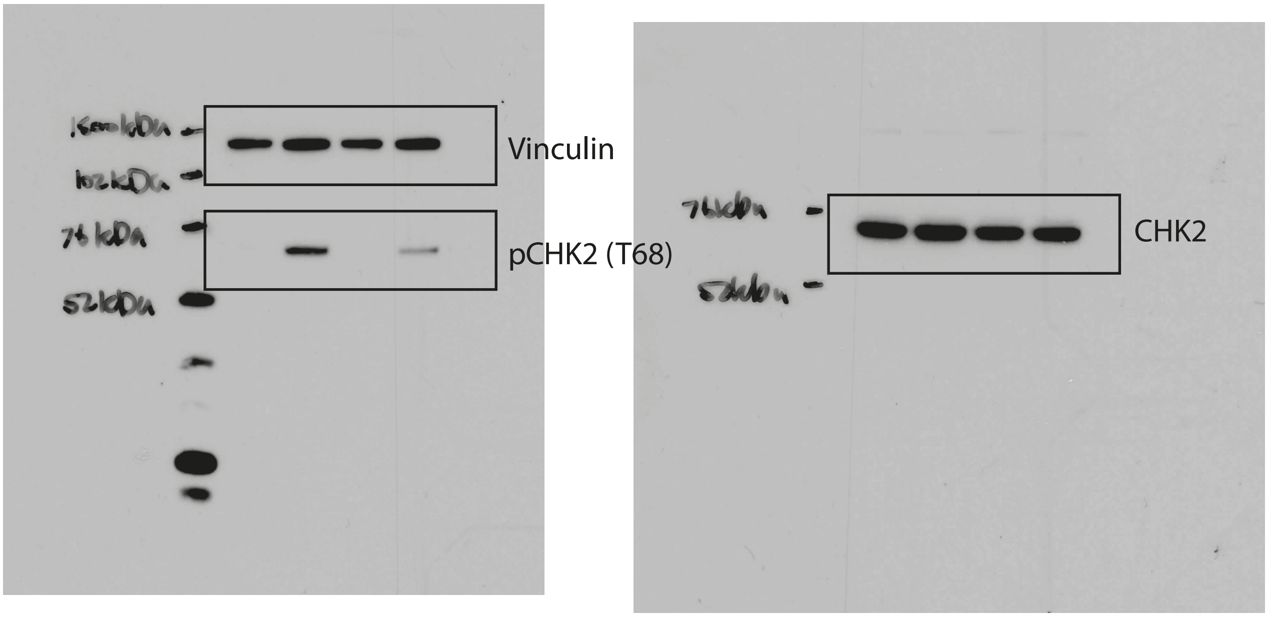

Figure 6b

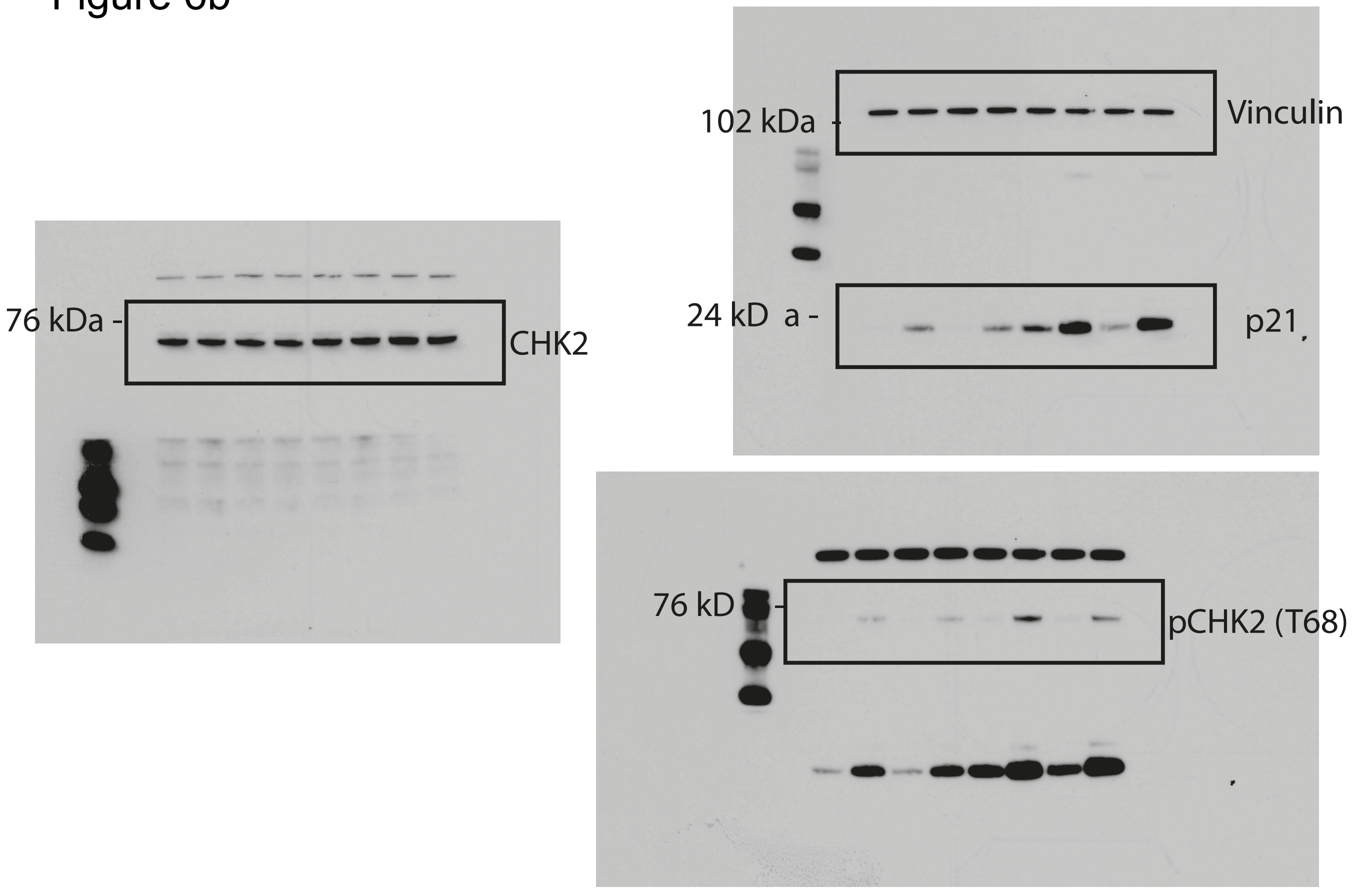

Extended Data Fig. 9c

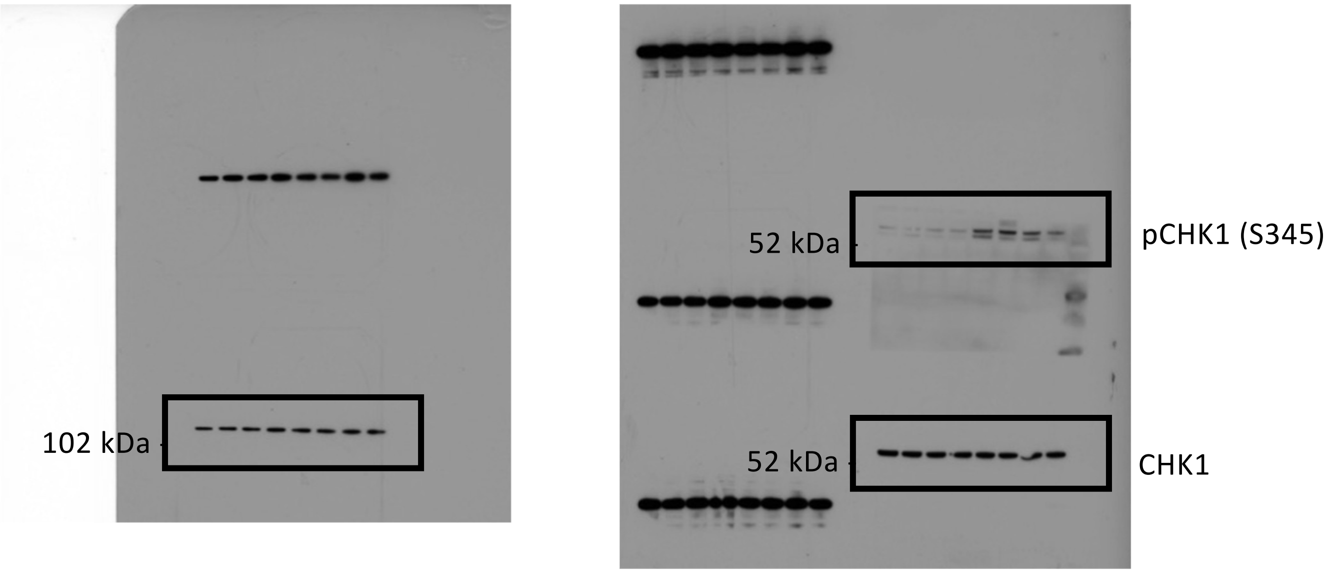

Extended Data Fig. 9e

pCHK2 (T68)

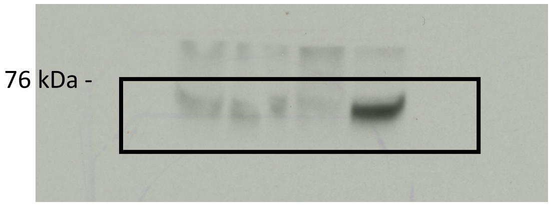

β-actin

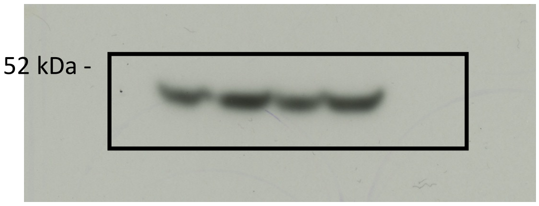

Extended Data Fig. 9f

pATM (S1981)

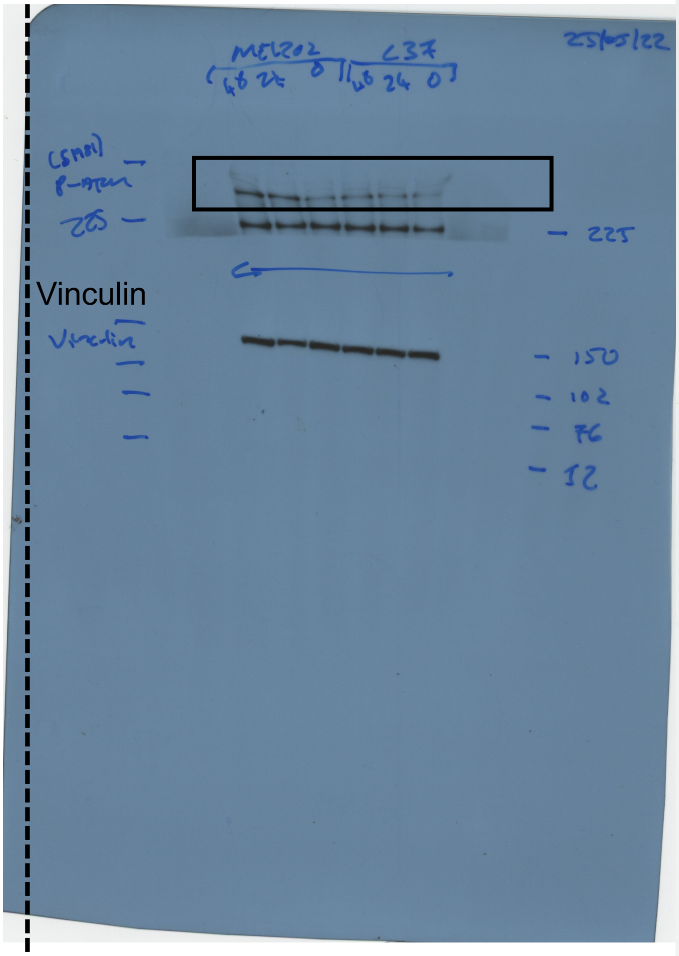

pATM (S1981)

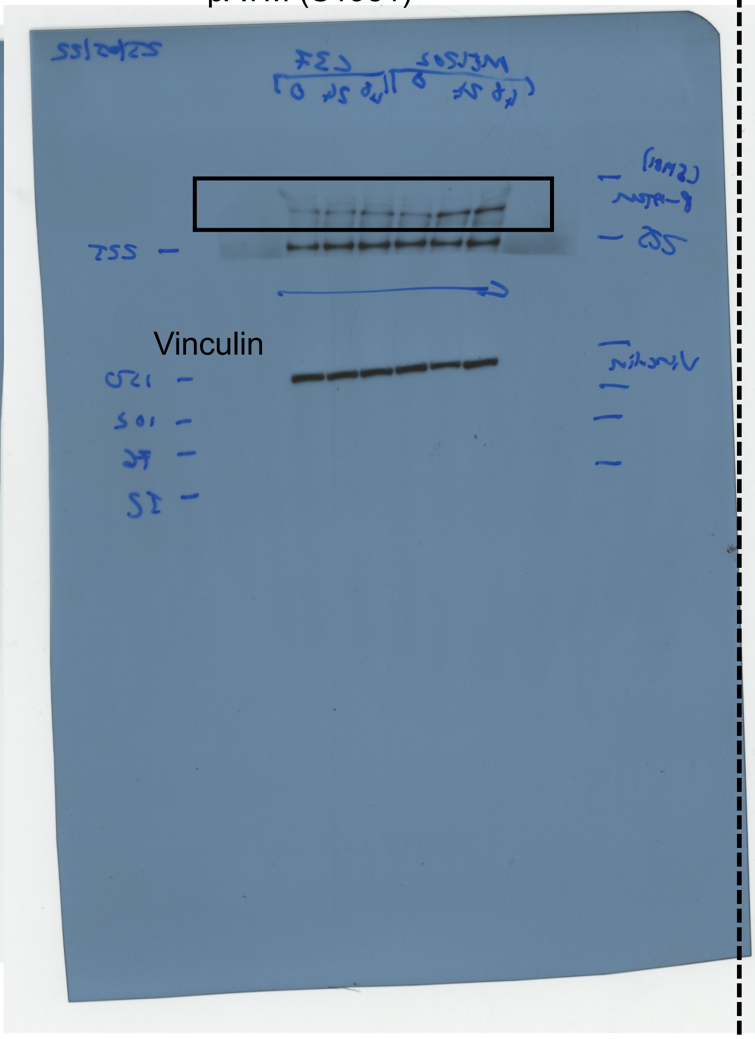

Extended Data Fig. 10d

pCHK2 (T68)

76 kDa -

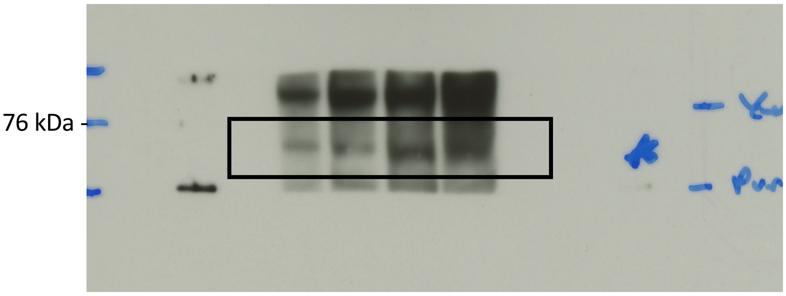

β-actin

52 kDa -

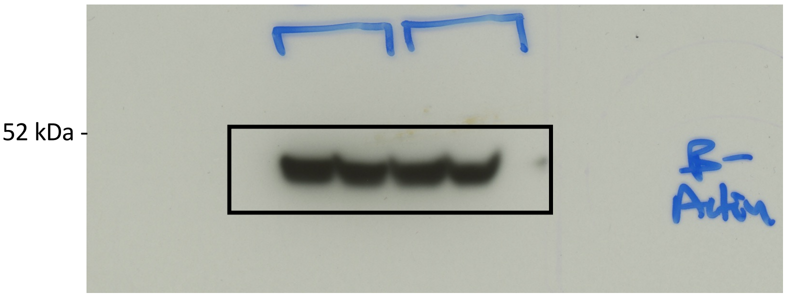

p21

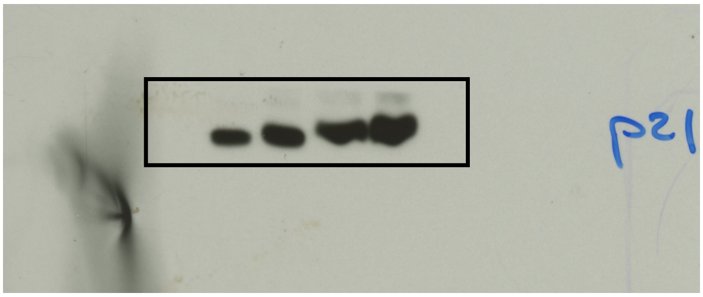

FACs gating example

related to Figure 5a

MEL202^{R625G}_DMSO

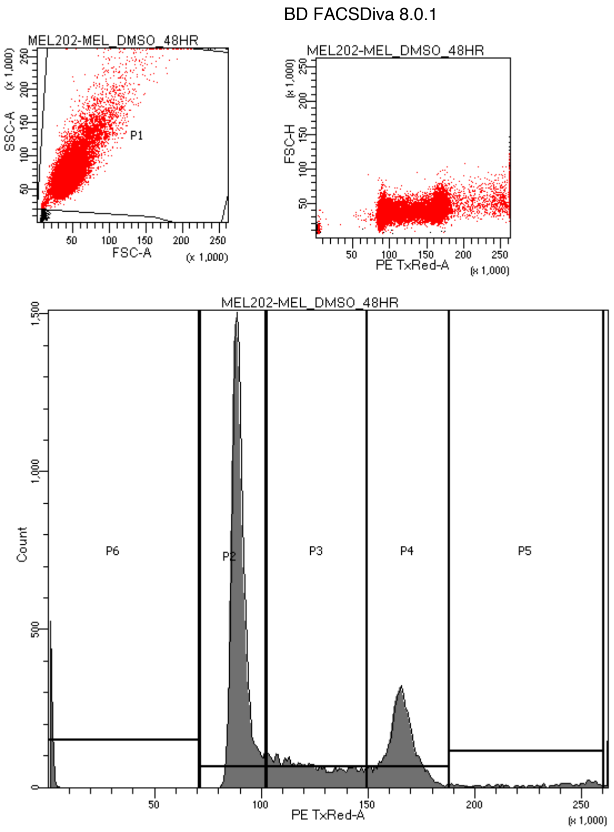

MEL202^{R625G}_PARPi

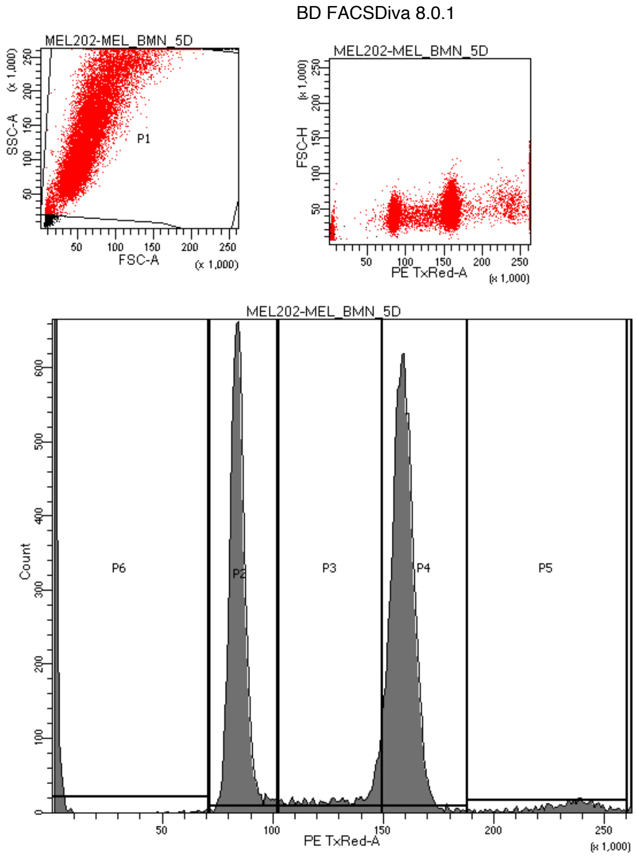

Fibre images

related to Figure 3a-d

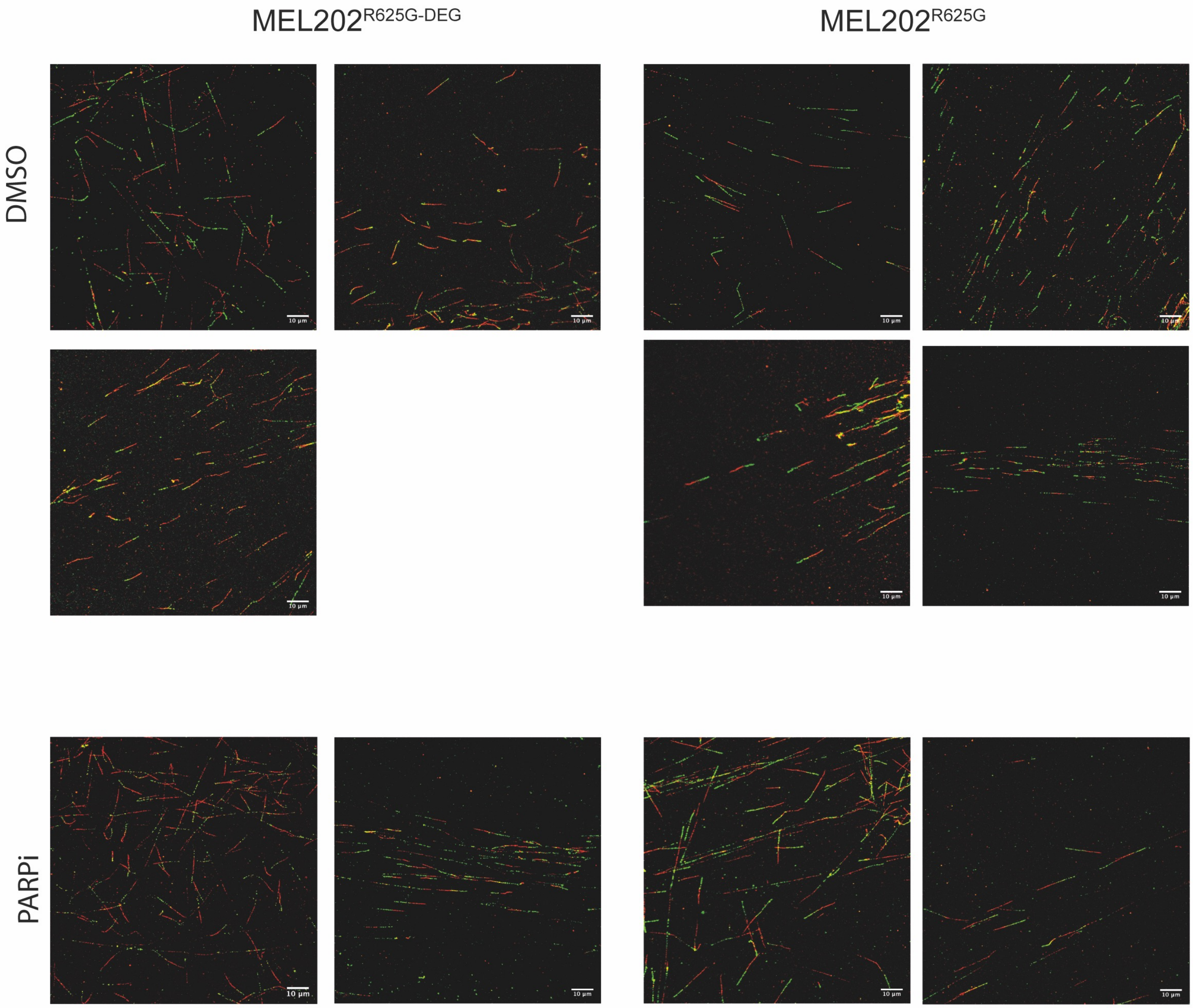

Fibre images

related to Figure 3a-d

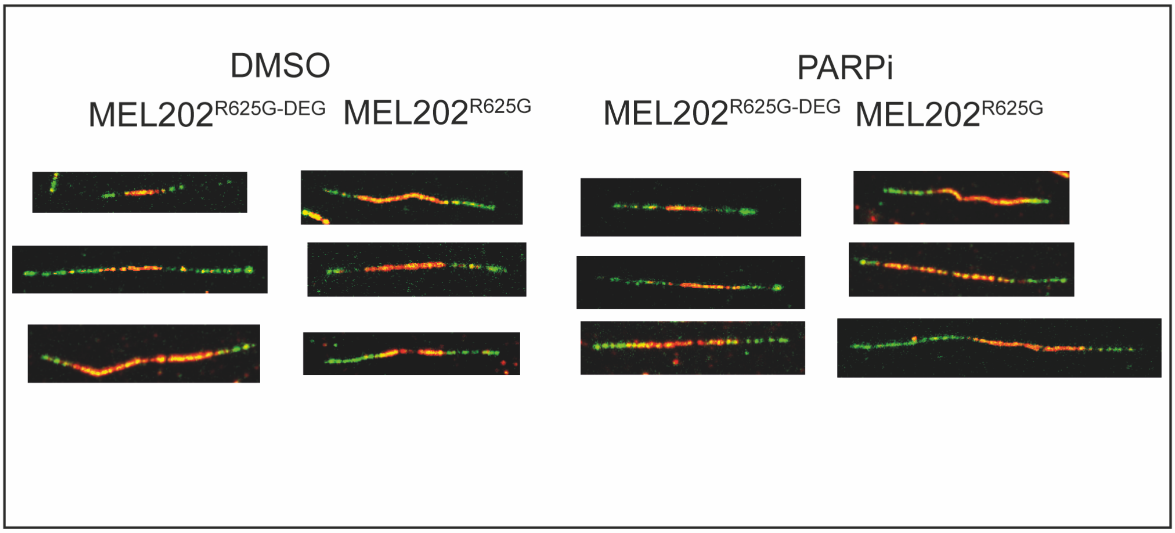

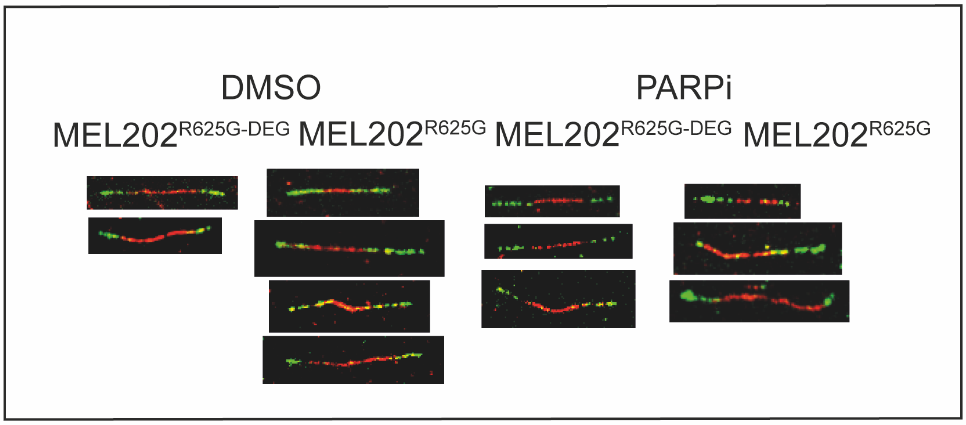

Supplement: Source Data Fig. 2–6 and Extended Data Figs. 1–6, 8–10 — Unmodified western blots—FACs gating images and uncropped DNA-fiber images. [file 41588_2023_1460_MOESM7_ESM.pdf]
